# Design and computational evaluation of *E*-stilbene-bearing 1,3,4-oxadiazole derivatives as potential tyrosinase inhibitors using DFT, molecular docking and ADMET studies

**DOI:** 10.1039/d6ra03363f

**Published:** 2026-07-07

**Authors:** Aasia Javed, Zulfiqar Ali Khan, Ameer Fawad Zahoor

**Affiliations:** a Department of Chemistry, Faculty of Physical Sciences, Government College University Faisalabad Faisalabad-38000 Pakistan zulfiqar.khan@gcuf.edu.pk

## Abstract

A series of *E*-stilbene-bearing 1,3,4-oxadiazole derivatives (4a–b and 5a–f) was synthesized *via* the Mizoroki–Heck reaction and evaluated as inhibitors of tyrosinase, a vital enzyme involved in melanin biosynthesis. The synthesized compounds were structurally confirmed through NMR spectroscopy (^1^H NMR, ^13^C NMR), infrared spectroscopy (FT-IR), and mass spectrometry. Nearly all derivatives exhibited inhibitory effects against tyrosinase with IC_50_ values ranging from 0.32 ± 0.03 µM to 6.28 ± 0.05 µM. Among them, compounds 5d and 5b illustrated the most significant inhibitory activity, with IC_50_ values of 0.32 ± 0.03 µM and 0.76 ± 0.01 µM, respectively. Molecular docking against tyrosinase (PDB: 2Y9X) revealed strong binding affinities (−10.0 kcal mol^−1^ and −8.5 kcal mol^−1^) exceeding those of the standard inhibitors kojic acid and ascorbic acid. Molecular dynamics simulation (100 ns), DFT analysis, and ADMET predictions further supported the stability, reactivity, and favorable pharmacokinetic behavior of the active compounds. These findings indicate that *E*-stilbene-based 1,3,4-oxadiazole derivatives hold significant potential as promising scaffolds for the development of new tyrosinase inhibitors for potential dermatological and cosmetic applications.

## Abstract 2

In this study, a series of *E*-stilbene-bearing 1,3,4-oxadiazole hybrids was designed and synthesized *via* Mizoroki–Heck reaction and evaluated as tyrosinase inhibitors. The most potent compounds were further subjected for *in silico* evaluation.

## Graphical abstract

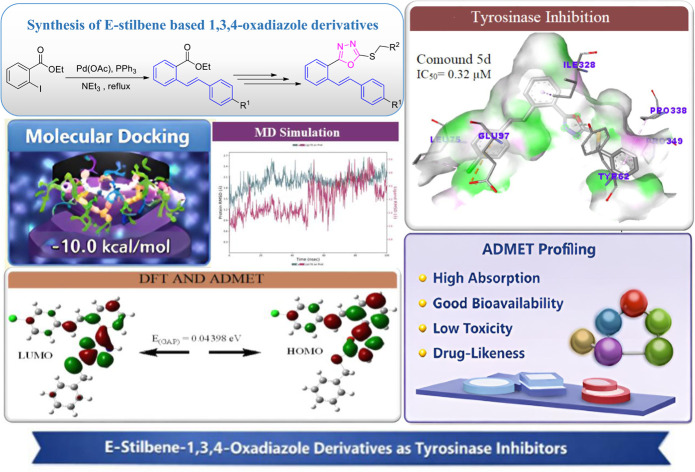

## 1. Introduction

Tyrosinase (EC 1.14.18.1) is a multifunctional copper-containing metalloenzyme that promotes the *ortho*-hydroxylation of monophenols to *o*-diphenols as well as oxidation of *o*-diphenols to *o*-quinones, using the oxidizing role of molecular oxygen.^1,2^ The enzyme contains a binuclear Cu active site, where each copper ion is linked to three conserved histidine residues, facilitating efficient activation of molecular oxygen for phenolic oxidation.^3^ As a member of the type-3 copper protein family, tyrosinase is recognized as a rate-limiting enzyme in the melanogenesis pathway and is ubiquitously distributed across plants, bacteria, fungi, and animals.^4–7^ The enzymatic activity of tyrosinase is integral to a range of biological processes, including pigmentation, plant defense responses, enzymatic browning in fruits and vegetables, and neuromelanin biosynthesis.^8^

In mammalian melanocytes, tyrosinase plays a central role in melanogenesis, promoting the conversion of l-tyrosine to l-3,4-dihydroxyphenylalanine (l-DOPA) and subsequently oxidizing l-DOPA to dopaquinone, ultimately leading to melanin biosynthesis.^9–11^ While melanin provides essential photoprotection against ultraviolet radiation, abnormal regulation or overexpression of tyrosinase may result in excessive melanin accumulation, thereby culminating in hyperpigmentation disorders like melasma, solar lentigines, and post-inflammatory hyperpigmentation.^12^

Beyond pigmentation, tyrosinase has also been implicated in neurodegenerative disorders, particularly Parkinson's disease, where it catalyzes the dopamine oxidation to dopamine *o*-quinone. This reaction affords reactive oxygen species (ROS), leading to oxidative stress, protein aggregation, and neuronal apoptosis that contribute to neurodegeneration.^8,13^ In plants, tyrosinase also known as polyphenol oxidase (PPO) is accountable for enzymatic browning in damaged tissues, which leads to quality deterioration and reduced shelf life of fruits and vegetables.^14–16^ Furthermore, in insects and arthropods, tyrosinase participates in immune defense, wound healing, and cuticle sclerotization through melanogenesis.^17^

Given its significant roles in pigmentation disorders, neurodegeneration, food spoilage, and insect physiology, tyrosinase has emerged as an important molecular target in cosmetic, pharmaceutical, and agricultural research. Consequently, substantial efforts have been focused on the discovery and development of new tyrosinase inhibitors with improved efficacy and safety profiles.^18^

Tyrosinase inhibition demonstrates a significant therapeutic and industrial strategy for the management of hyperpigmentation disorders, mitigation of oxidative stress associated with neurodegenerative diseases, prevention of enzymatic browning in food products, and control of insect pests.^2,3,6^ A variety of natural phenolic and flavonoid compounds, including kojic acid, ascorbic acid, kaempferol, glabrene, cuminaldehyde, and arbutin, have been extensively reported as potent tyrosinase inhibitors. However, their practical clinical and commercial applications are limited due to inherent drawbacks such as poor chemical stability, low bioavailability, inadequate skin permeability, and undesirable adverse effects, including dermatitis, cytotoxicity, and potential cutaneous complications.^19,20^ These limitations have consequently stimulated the rational design and development of structurally optimized synthetic tyrosinase inhibitors with improved efficacy, safety, and pharmacokinetic profiles.

**Fig. 1:**
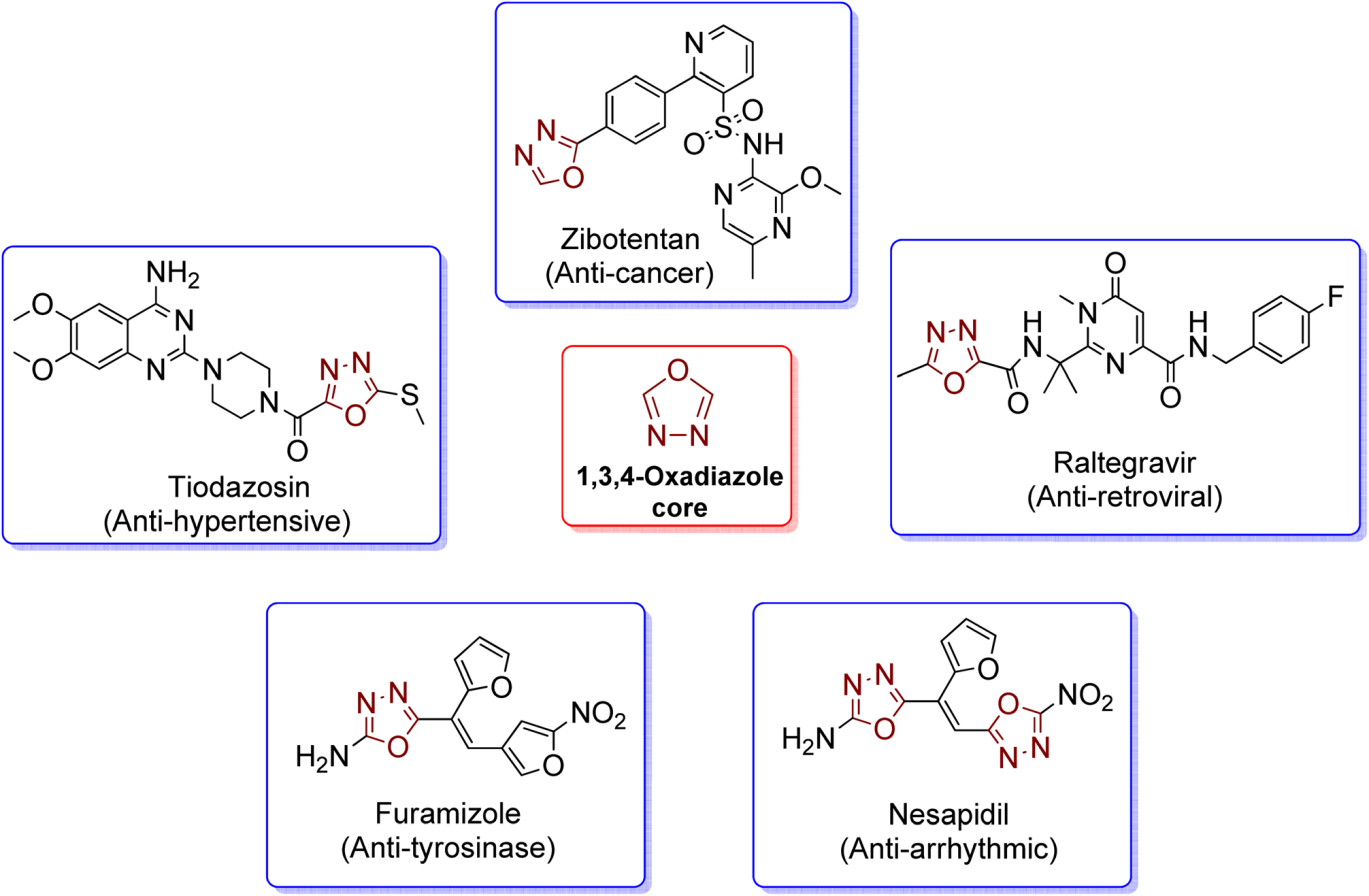
Representative examples of drugs incorporating the 1,3,4-oxadiazole core structure.

Among synthetically accessible compounds, heterocyclic scaffolds such as triazoles, coumarins, thiadiazoles, quinoxaline-sulfonamides, and quinolones have demonstrated promising anti-tyrosinase activity. In particular, compounds containing azole moieties exhibit enhanced inhibitory potential against tyrosinase.^21–25^ Within this class, oxadiazoles five-membered heterocyclic rings containing one oxygen, two nitrogen, and two carbon atoms have attracted considerable attention. Among the different isomers, 1,3,4-oxadiazole derivatives have been shown to possess strong tyrosinase inhibitory activity^25^ as well as other biologically relevant enzymes, including urease,^26^ acetylcholinesterase,^27^ and carbonic anhydrase.^28^ In addition, numerous 1,3,4-oxadiazole-based compounds possess a wide range of pharmacological activities, such as antiviral, antihypertensive, antibacterial, anticancer, antiarrhythmic, and antiretroviral effects^29,30^ (Fig. 1). Consequently, the oxadiazole scaffold has emerged as an important pharmacophore in modern drug discovery and development.^31^

The ‘stilbene family’ belongs to an important class of naturally occurring biologically active compounds that exhibit significant inhibition against tyrosinase.^32^ Resveratrol (*trans*-3,5,4′-trihydroxystilbene), commonly found in berries, red wine, grapes and berries, exhibits various biological activities including anti-tyrosinase, anticancer, anti-inflammatory, and antioxidant properties.^33^ Among stilbene derivatives, resveratrol (3,5,4′-trihydroxy-*trans*-stilbene) and oxyresveratrol (2,3′,4,5′-tetrahydroxy-*trans*-stilbene) are well-known tyrosinase inhibitors belonging to the *E*-stilbene class.^16,34^ However, despite their promising biological potential, these compounds exhibit relatively weak inhibition toward diphenolase activity, which limits their effectiveness as anti-browning and skin-depigmenting agents.^35^ Consequently, significant research efforts have focused on the synthesis of more promising stilbene-derivatives with improved tyrosinase inhibitory activity.

Despite extensive research, an ideal tyrosinase inhibitor that combines high potency, safety, and chemical stability remains elusive. This limitation underscores the need for developing novel compounds with promising pharmacological profiles. In recent years, molecular hybridization has emerged as an effective strategy in rational drug design, allowing the integration of multiple bioactive pharmacophores within a single molecular framework to augment their biological activity, selectivity, and physicochemical properties.^36^

Stilbene derivatives exhibit significant biological activities, including antioxidant, anticancer, and enzyme inhibitory properties, while 1,3,4-oxadiazole represents an important heterocyclic scaffold owing to its wide pharmacokinetic profile and broad spectrum of biological activities. Therefore, the hybridization of stilbene and oxadiazole motifs may provide a promising platform for developing novel tyrosinase inhibitors. Thus, the current study reports the design and synthesis of *E*-stilbene-bearing oxadiazole conjugates and evaluates their potential as tyrosinase inhibitors through biological assays supported by molecular docking, DFT calculations, and ADMET analysis.

## 2. Experimental

### 2.1. Chemicals and instruments

All chemicals and solvents were obtained from commercial suppliers and utilized without additional purification. The reactions were carried out in dry glassware under suitable experimental conditions. To confirm the reaction completion, TLC was conducted. Purification of the products was achieved through column chromatography using silica gel (70–230 mesh). Nuclear magnetic resonance (NMR) spectra were obtained *via* Bruker Avance III HD 400 MHz spectrometer employing deuterated chloroform (as solvent) and tetramethylsilane (TMS) (as internal standard). Chemical shift values (*δ*), signal multiplicities, and coupling constants (*J*) were analyzed from the acquired FID data. Fourier-transform infrared spectra were obtained *via* Agilent Cary 630 FT-IR spectrometer and mass spectrometric analysis were conducted on a TSQ Quantis Triple Quadrupole Mass Spectrometer.

#### 2.1.1 General synthetic protocol

The synthesis of compounds 2a–b, 3a–b and 4a–b was carried out *via* reported protocol.^37^ To accomplish the synthesis of compounds 5a–f, corresponding 1,3,4-oxadiazole-2-thiol (4a or 4b) (3 mmol) was dissolved in DMF, and potassium carbonate (6 mmol) was added to resulting mixture followed by the addition of acyl bromides (3 mmol). After stirring the reaction mixture at room temperature for 2–4 hours, TLC was carried out to monitor reaction progress. Upon completion, ice-cold water was poured into reaction mixture, leading to the formation of white precipitates. These were collected by filtration, washed thoroughly with water. The dried precipitates were then finally recrystallized using an appropriate solvent to attain the purified product.

#### 2.1.2 (*E*)-2-(Isopentylthio)-5-(2-styrylphenyl)-1,3,4-oxadiazole (5a)

Compound 5a was synthesized by reacting equimolar amounts of 4a (3 mmol) with isopentylbromide (3 mmol) according to general synthetic procedure. Yield: 59%, white solid; m.p. 72–73 °C; *R*_f_: 0.50; ^1^H NMR (CDCl_3_, 400 MHz): 7.85 (1H, d, *J* = 8.0 Hz), 7.41–7.14 (10H, m), 2.91 (2H, t, *J* = 8.0 Hz), 1.80–1.70 (3H, m), 0.96 (6H, d, *J* = 8.0 Hz); ^13^C NMR (CDCl_3_, 100 MHz): 165.47, 164.29, 142.98, 141.85, 141.57, 131.25, 131.17, 129.08, 128.62, 128.26, 126.46, 125.87, 124.85, 122.42, 37.99, 30.69, 27.42, 22.14. FT-IR (*ν*_max_, cm^−1^): 2924, 1599, 1476, 1182, 1036, 963, 804, 710, 534; ESI-MS calcd for C_21_H_22_N_2_OS: 349.2 [M–H]^−^.

#### 2.1.3 (*E*)-2-(2-(4-Chlorostyryl)phenyl)-5-(isopentylthio)-1,3,4-oxadiazole (5b)

The compound 5b was synthesized by reacting equimolar amounts of 4b (3 mmol) with isopentylbromide (3 mmol) according to general synthetic procedure. Yield: 64%, white solid; m.p. 60–61 °C; *R*_f_: 0.75; ^1^H NMR (CDCl_3_, 400 MHz): 8.22 (1H, d, *J* = 8.0 Hz), 7.78–7.88 (2H, m), 7.51–7.49 (3H, m), 7.39–7.30 (3H, m), 7.04 (1H, d, *J* = 16.0 Hz), 3.31 (2H, t, *J* = 8.0 Hz), 1.79–1.69 (2H, m), 1.24 (1H, broad s), 0.95 (6H, d, *J* = 8.0 Hz); ^13^C NMR (CDCl_3_, 100 MHz): 165.41, 164.52, 137.08, 135.73, 133.61, 131.30, 130.71, 129.00, 128.84, 128.23, 127.74, 127.51, 126.76, 121.46, 37.93, 30.69, 27.41, 22.12; FT-IR (*ν*_max_, cm^−1^): 2940, 2330, 1599, 1464, 1190, 1040, 750, 689; ESI-MS calcd for C_21_H_21_ClN_2_OS: 383.2[M–H]^−^.

#### 2.1.4 (*E*)-2-(Benzylthio)-5-(2-styrylphenyl)-1,3,4-oxadiazole (5c)

The compound 5c was synthesized by reacting equimolar amounts of 4a (3 mmol) with benzyl bromide (3 mmol) according to general synthetic procedure. Yield: 71%, white crystalline solid; m.p. 96–97 °C; *R*_f_: 0.40; ^1^H NMR (CDCl_3_, 400 MHz): 8.19 (1H, d, *J* = 16.0 Hz), 7.85–7.78 (2H, m), 7.50–7.44 (6H, m), 7.39–7.29 (6H, m), 7.04 (1H, d, *J* = 16.0 Hz); 4.54 (2H, s); ^13^C NMR (CDCl_3_, 100 MHz): 165.60, 163.84, 137.13, 135.70, 135.43, 133.64, 131.38, 130.77, 129.11, 129.05, 128.84, 128.84, 128.23, 128.14, 127.74, 127.44, 126.77, 121.36, 36.78; FT-IR (*ν*_max_, cm^−1^): 3065, 2930, 1490, 1200, 978, 776, 711; ESI-MS calcd for C_23_H_18_N_2_OS: 369.3 [M–H]^−^.

#### 2.1.5 (*E*)-2-(Benzylthio)-5-(2-(4-chlorostyryl)phenyl)-1,3,4-oxadiazole (5d)

The compound 5d was synthesized by reacting equimolar amounts of 4b (3 mmol) with benzyl bromide (3 mmol) according to general synthetic procedure. Yield: 78%, white crystalline solid; m.p. 102–103 °C; *R*_f_: 0.50; ^1^H NMR (CDCl_3_, 400 MHz): 8.18 (1H, d, *J* = 16.0 Hz); 7.85–7.81 (2H, m), 7.57 (2H, d, *J* = 8.0 Hz), 7.51–7.44 (3H, m), 7.37–7.24 (6H, m), 7.11 (1H, d, *J* = 16.0 Hz); 4.54 (2H, s); ^13^C NMR (CDCl_3_, 100 MHz): 165.71, 163.78, 137.49, 137.16, 135.46, 132.19, 131.35, 129.12, 129.07, 128.83, 128.68, 128.12, 128.03, 127.52, 127.07, 126.80, 126.75, 121.35, 36.79; FT-IR (*ν*_max_, cm^−1^): 3082, 1552, 1482, 1202, 1019, 974, 784, 715, 685; ESI-MS calcd for C_23_H_17_ClN_2_OS: 403.8 [M–H]^−^.

#### 2.1.6 (*E*)-2-(Octylthio)-5-(2-styrylphenyl)-1,3,4-oxadiazole (5e)

The compound 5e was synthesized by reacting equimolar amounts of 4a (3 mmol) with *n*-octyl bromide (3 mmol) according to general synthetic procedure. Yield: 59%, white crystalline solid; m.p. 58–59 °C; *R*_f_: 0.71 ^1^H NMR (CDCl_3_, 400 MHz): 8.21 (1H, d, *J* = 16.0 Hz), 7.88 (1H, d, *J* = 8.0 Hz), 7.82 (1H, d, *J* = 8.0 Hz), 7.57 (2H, d, *J* = 8.0 Hz), 7.49 (1H, d, *J* = 8.0 Hz), 7.38–7.33 (3H, m), 7.27 (1H, d, *J* = 8.0 Hz), 7.10 (1H, d, *J* = 16.0 Hz) 3.29 (2H, t, *J* = 8.0 Hz), 1.87–1.81 (2H, m), 1.48–1.41 (2H, m) 1.32–1.21 (8H, m) 0.86 (3H, t, *J* = 8.0 Hz); ^13^C NMR (CDCl_3_, 100 MHz): 165.52, 164.49, 137.43, 137.18, 132.11, 131.26, 129.03, 128.66, 128.00, 127.51, 127.06, 126.83, 126.77, 121.45, 32.58, 31.73, 29.24, 29.09, 28.97, 28.61, 22.59, 14.06; FT-IR (*ν*_max_, cm^−1^): 3061, 2919, 2856, 1480, 1211, 1054, 974, 769, 717; ESI-MS calcd for C_24_H_28_N_2_OS: 391.1 [M–H]^−^.

#### 2.1.7 (*E*)-2-(2-(4-Chlorostyryl)phenyl)-5-(octylthio)-1,3,4-oxadiazole (5f)

The compound 5f was synthesized by reacting equimolar amounts of 4b (3 mmol) with *n*-octyl bromide (3 mmol) according to general synthetic procedure. Yield: 63%; colorless crystalline solid; m.p. 74–75 °C; *R*_f_: 0.61. ^1^H NMR (CDCl_3_, 400 MHz): 8.20 (1H, d, *J* = 16.0 Hz), 8.87 (1H, d, *J* = 8.0 Hz), 7.79 (1H, d, *J* = 8.0 Hz), 7.50–7.48 (3H, m), 7.39–7.29 (3H, m), 7.04 (1H, d, *J* = 16.0 Hz), 3.29 (2H, t, *J* = 8.0 Hz), 1.87–1.79 (2H, m), 1.48–1.42 (2H, m), 1.25–1.21 (8H, m), 0.86 (3H, t, *J* = 8.0 Hz); ^13^C NMR (CDCl_3_, 100 MHz): 165.41, 164.58, 137.06, 135.72, 133.60, 131.30, 130.69, 129.00, 128.83, 128.21, 127.74, 127.49, 126.74, 121.45, 32.57, 31.73, 29.23, 29.09, 28.97, 28.61, 22.59, 14.05; FT-IR (*ν*_max_, cm^−1^): 3074, 2932, 2858, 1482, 1198, 1104, 1021, 980, 834, 728. ESI-MS calcd for C_24_H_27_ClN_2_OS: 425.5 [M–H]^−^.

### 2.2. Biological evaluation

The anti-tyrosinase potential of synthesized *E*-stilbene based oxadiazole derivatives was studied using spectrophotometric assay utilizing reported method.^38^ The tyrosine enzyme was isolated from *Agaricus bisporus*. The synthesized styrene-linked oxadiazole derivatives were assessed for their ability to inhibit tyrosinase by employing l-DOPA as the substrate. For this purpose, 2.0% solution of the all the synthesized derivatives was obtained by dissolving them in DMSO. Next, the concentrated stock solution was mixed with phosphate buffer (at a pH of 6.8). The pre-incubation of compounds and tyrosine enzyme was done for 10 min at room temperature, followed by the incorporation of levodopa (0.5 mM). The absorbance analysis of reaction mixture was done at 474 nm. The IC_50_ values were measured by estimating dose–response curve and the percentage inhibition was calculated usingPercentage inhibition = (*B* − *S*/*B*) × 100where *B* and *S* correspond to absorbance values for the blank and sample, correspondingly. Ascorbic acid was used as standard drug.

### 2.3. Docking studies

The oxadiazole derivatives 5d and 5b were selected for the molecular docking studies against anti-tyrosinase with PDB: 2Y9X.^39^ The crystal structure of *Agaricus bisporus* tyrosinase (PDB ID: 2Y9X) has been widely employed as a representative and reliable model in molecular docking and simulations studies of tyrosinase inhibitors. The tyrosinase protein's crystalline structure was downloaded from the protein data bank. The protein preparation was done using BIOVIA Discovery Studio 2024. The ligands and water molecules were removed and it was saved in PDB format. The saved protein file was opened in AutoDock Vina 1.5.7 and Kollman charges and polar hydrogens were incorporated.^40^ The suitable dimensions of axis and size were adjusted using the grid box. After the completion of docking analysis, the poses for each ligand were saved and the results for the ligand–protein complex were analyzed using BIOVIA Discovery Studio 2024.

### 2.4. MD simulation studies

The potent compounds *i.e.*, 5d and 5b were further subjected to molecular dynamics simulation analysis for ligand–protein interactions. The MD simulations were performed using Maestro's Academic version of Schrödinger (Desmond module).^41^ The preparation of protein–ligand complex was done utilizing the preparation wizard of the Maestro followed by the introduction of an orthorhombic box and TIP3P solvent model *via* the system builder wizard. The prepared protein was first saved and then opened again in the Maestro for the molecular dynamics studies. The simulation time was adjusted to 100 ns at 1 atm and 310 K temperature. After the completion, SID (Simulation Interaction Diagram) feature was employed for the result visualization.^42^

### 2.5. DFT analysis

The potent compound 5d was also subjected for DFT analysis to obtain its optimized molecular structure and to elucidate the reactivity and molecular properties. Gaussian 09W and Gaussview were the software programs employed to perform DFT calculations at B3LYP/6-31-G basis set.^43^ The HOMO–LUMO energies, Mulliken atomic charges, molecular electrostatic potential (MEP), and their images were extracted and evaluated using Gaussview software.

### 2.6. ADME and related studies

The synthesized derivatives of oxadiazole were evaluated for their ADME and other physiochemical properties, such as toxicokinetic and medicinal profile. The ADMET analysis was done using ADMETLab 3.0,^44^ which is an easily accessible online tool. The structures for all the synthesized derivatives were transported from ChemDraw Professional 16.0 in the form of SMILES.

## 3. Results and discussion

### 3.1. Chemistry

**Scheme 1:**
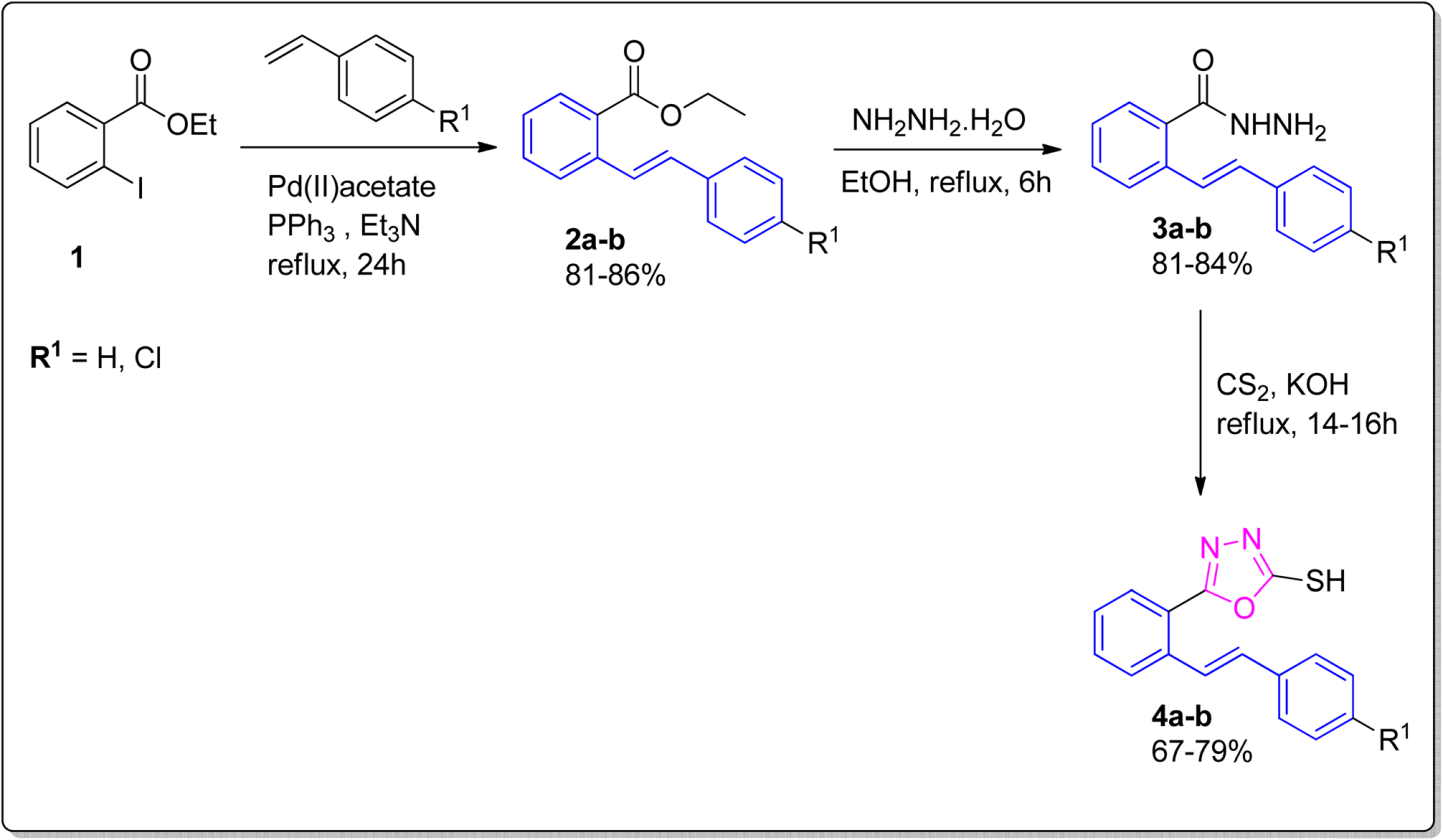
Synthesis of *E*-stilbene bearing oxadiazole hybrids (4a–b) *via* the Mizoroki–Heck reaction.

**Scheme 2:**
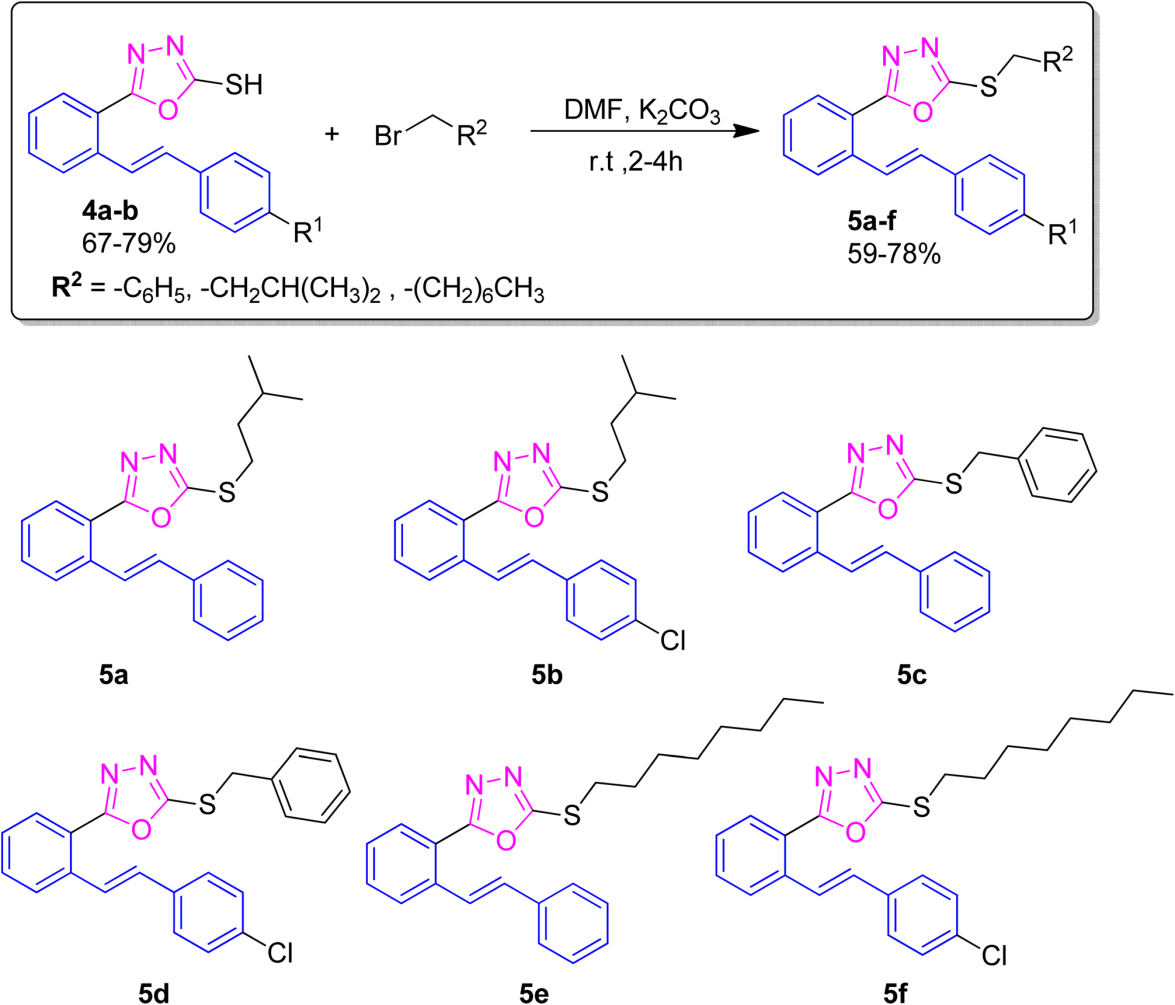
Synthesis of 2,5-disubstituted *E*-stilbene bearing oxadiazole hybrids (5a–f).

The designed synthetic framework for the preparation of *E*-stilbene-based 1,3,4-oxadiazole derivatives is illustrated in Schemes 1 and 2. Initially, *E*-stilbene esters (2a–b) were synthesized utilizing Mizoroki–Heck reaction, by coupling 2-iodophenyl ethyl ester 1 with styrene or 2-chlorostyrene using catalytic role of palladium acetate (Pd(OAc)_2_).^37,45,46^ The corresponding hydrazide intermediates (3a–b) were obtained by refluxing esters (2a–b) with an excess of hydrazine monohydrate in ethanol. Subsequent cyclization of hydrazides (3a–b) was carried out with carbon disulfide (CS_2_) in the presence of potassium hydroxide (KOH) as per reported protocol,^37,47^ leading to the formation of *E*-stilbene bearing oxadiazole hybrids (4a–b) (Scheme 1).

Finally, the targeted *E*-stilbene-based oxadiazole derivatives (5a–f) were synthesized *via* the reaction of oxadiazole-2-thiols (4a–b) with various alkyl halides in the presence of potassium carbonate (K_2_CO_3_) (Scheme 2).

### 3.2. Anti-tyrosinase activity

The synthesized *E*-stilbene bearing oxadiazole hybrids (4a–b and 5a–f) were evaluated for their potential as tyrosinase inhibitors. The results demonstrated that all compounds illustrated efficient inhibitory activity against the tyrosinase enzyme compared to the standards, namely kojic acid (IC_50_ = 30.34 ± 0.75 µM) and ascorbic acid (IC_50_ = 11.5 ± 1.00 µM).^48^

Among the tested derivatives, compounds 5b and 5d showed the most promising activity with 0.76 ± 0.01 µM and 0.32 ± 0.03 µM IC_50_ values, respectively, along with percentage inhibition values of 78.27% and 68.58%. Additionally, compounds 5e and 5f also demonstrated strong anti-tyrosinase activity, with 1.43 ± 0.02 µM and 1.18 ± 0.01 µM IC_50_ values, and corresponding inhibition percentages of 74.54% and 75.21%, respectively.

Furthermore, compounds 5a, 4a, and 4b exhibited moderate inhibitory activity with of 2.2 ± 0.05 µM, 4.51 ± 0.09 µM, and 4.2 ± 0.10 µM IC_50_ values, correspondingly. Although their activity was lower compared to compounds 5b, 5d, 5e, and 5f, they still exhibited higher potency than the standard inhibitors. Moreover, compound 5c exhibited the lowest tyrosinase inhibitory potential among all derivatives with IC_50_ values of 6.28 ± 0.05 µM; however, it still demonstrated superior potency compared to the standard drugs (Table 1).

**Table 1:** IC_50_ values and %age inhibition of synthesized derivatives (4a–b and 5a–f) against tyrosinase enzyme, in comparison with reference standards

Sr. no.	Compounds	Structure	IC_50_ (µM) ± S. E.	Percentage inhibition (%)
1	4a	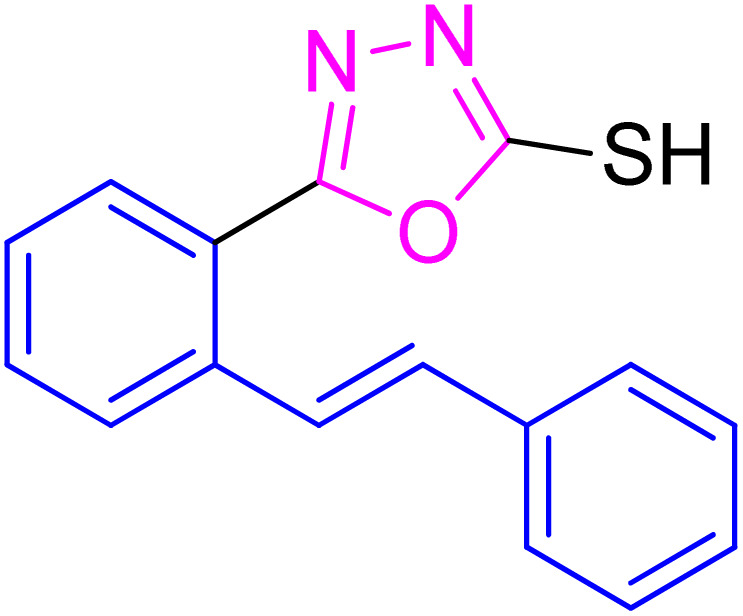	4.51 ± 0.09	96.29
2	4b	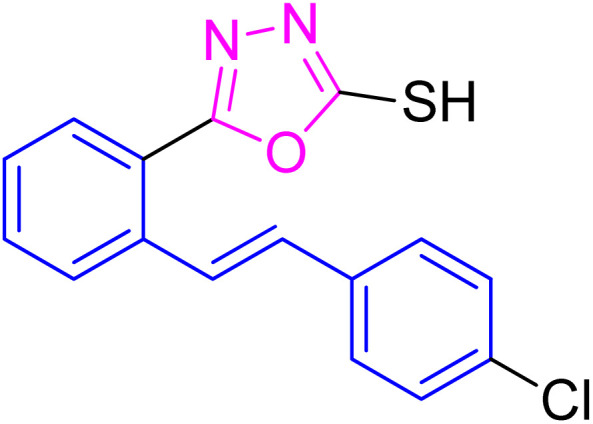	4.2 ± 0.10	88.98
3	5a	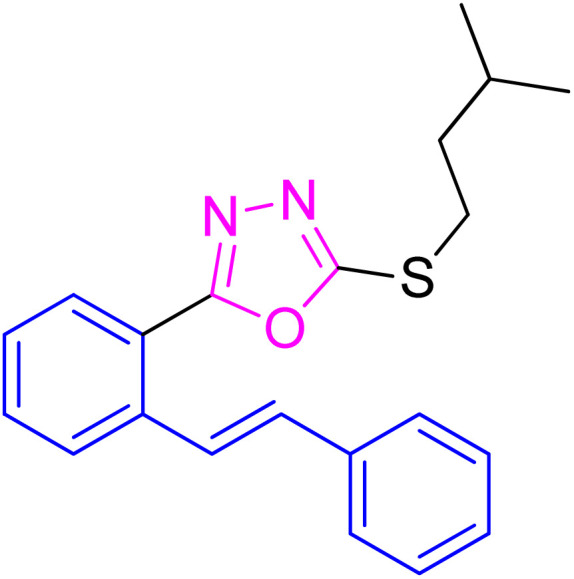	2.2 ± 0.05	54.62
4	5b	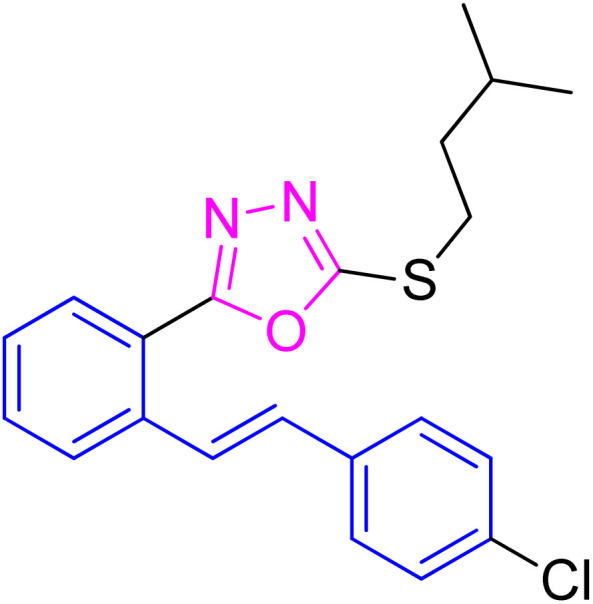	0.76 ± 0.01	78.27
5	5c	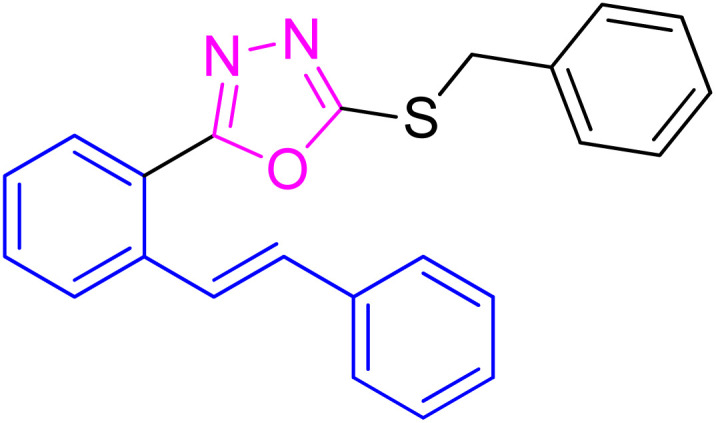	6.28 ± 0.05	63.61
6	5d	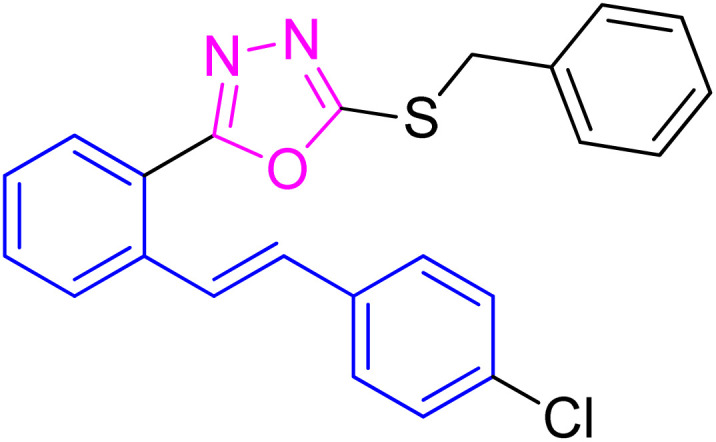	0.32 ± 0.03	68.58
7	5e	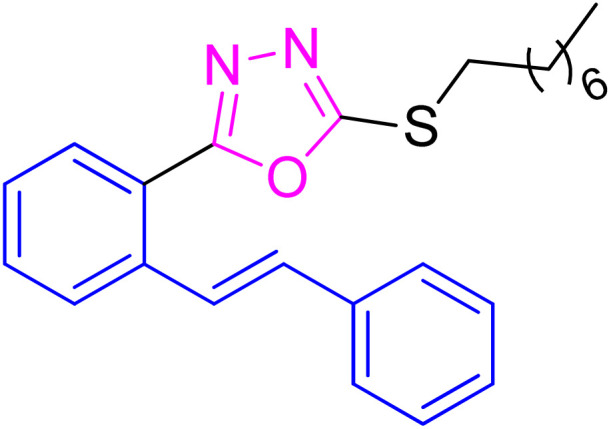	1.43 ± 0.02	74.54
8	5f	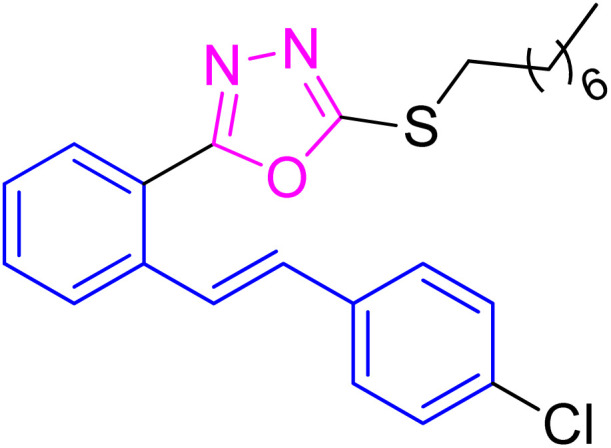	1.18 ± 0.01	75.21
9	Kojic acid	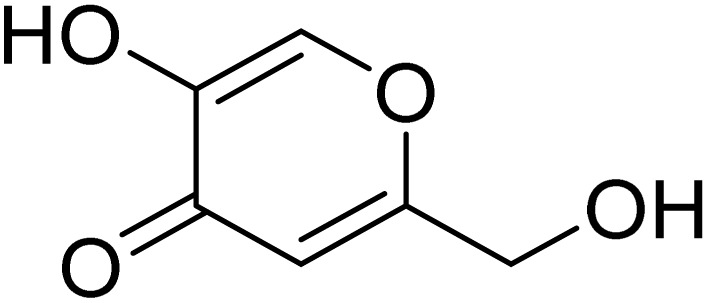	30.34 ± 0.75	6.80 ± 0.58
10	Ascorbic acid	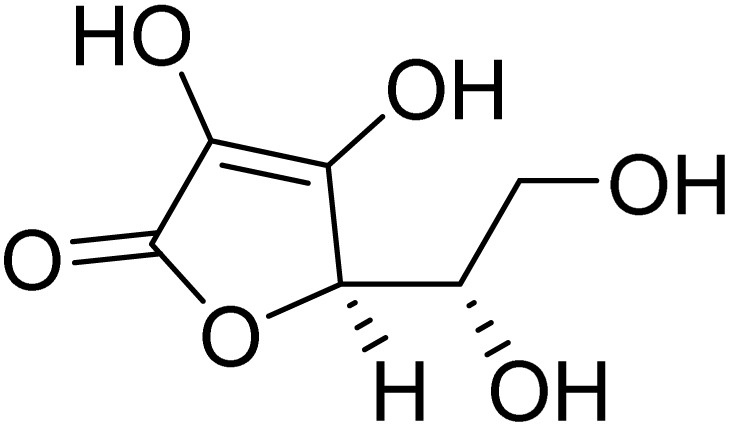	11.5 ± 1.00	58.66 ± 1.00

#### 3.2.1 Structure–activity relationship (SAR)

SAR analysis inferred that both the nature of substituents and hydrophobicity significantly influenced tyrosinase inhibitory activity. Among the analyzed hybrids, compounds substituted with halogen groups (*e.g.*, 5b and 5d) exhibited enhanced activity, which may be attributed to improved π–anion and hydrophobic interactions within catalytic site of enzyme. In particular, the presence of chloro-substituent facilitated stronger binding interactions with key residues such as GLU-97 and TYR-62, as evidenced by docking studies.

Furthermore, derivatives containing benzylthio-substitution (5d) demonstrated superior activity compared to alkyl-substituted analogues (5a, 5e), suggesting that increased aromatic character enhances π–π stacking and stabilizes ligand–protein interactions. These findings were further supported by the comparatively lower activity of the unsubstituted analogue 5c, indicating the favorable contribution of the chloro group toward enhanced anti-tyrosinase activity.

### 3.3. *In silico* studies

#### 3.3.1 Docking analysis

The most potent *E*-stilbene-based oxadiazole derivatives, 5b and 5d, exhibiting promising anti-tyrosinase activity, were selected for molecular docking studies to elucidate ligand–protein interactions. The crystal structure of *A. bisporus* tyrosinase (PDB ID: 2Y9X) was retrieved from the PDB (Protein Data Bank) and employed as the target protein. For comparative analysis, kojic and ascorbic acid were also docked under identical conditions.

The compounds were evaluated based on their lowest binding energies and interaction profiles, including hydrophilic and hydrophobic contacts contributing to stable binding within the catalytic pocket of the enzyme.

**Fig. 2:**
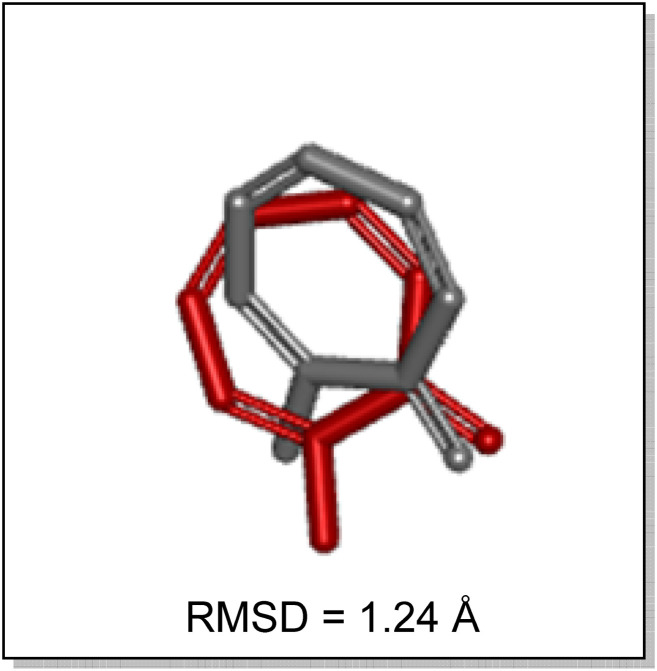
Validation of docking protocol by redocking of co-crystallized ligand (gray) and docked ligand (red).

Initially, docking protocol was validated by redocking the co-crystallized ligand within the enzyme catalytic center. The calculated RMSD (root mean square deviation) value between the docked and crystallographic ligand was 1.2428 Å, thereby indicating the precision of docking parameters and methodology (Fig. 2).

**Fig. 3:**
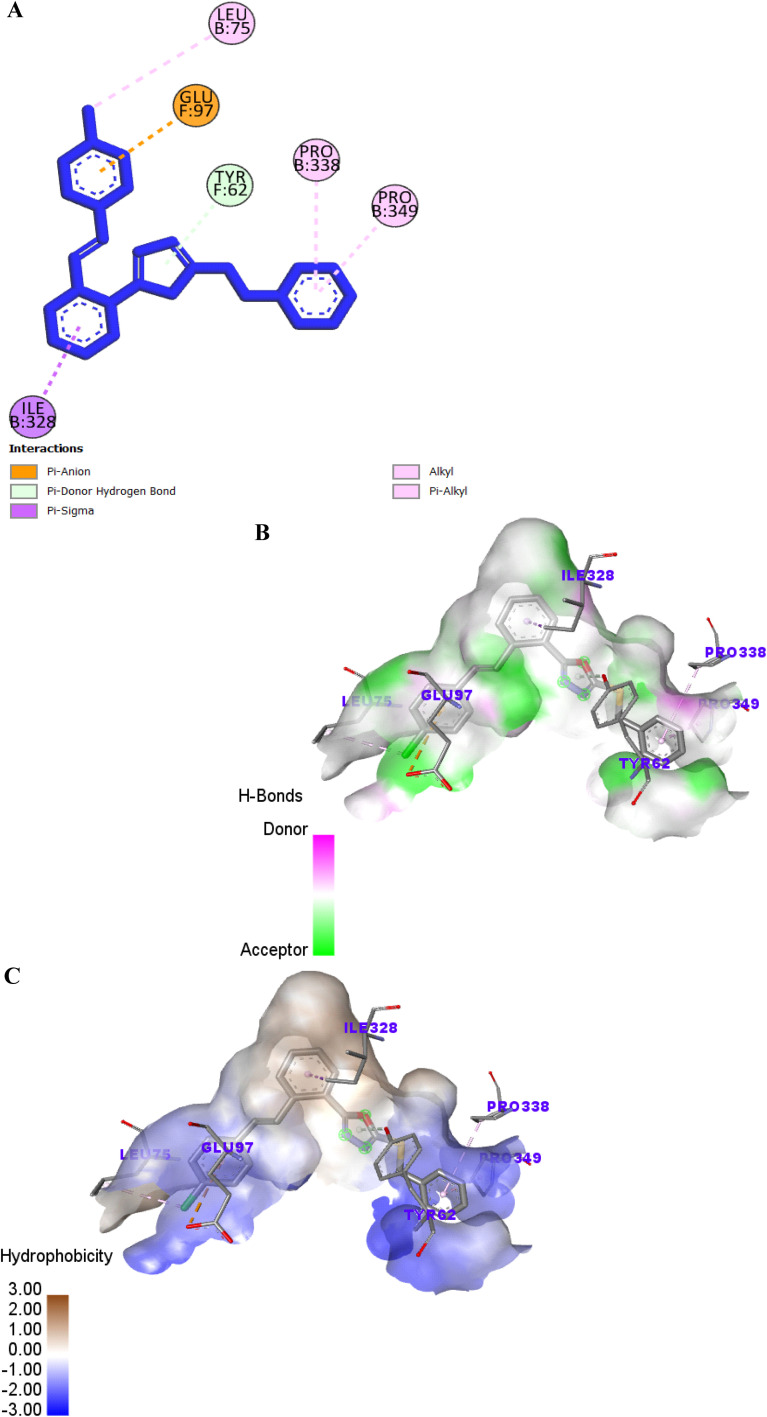
Docking studies of the synthesized compound 5d (A) 2D ligand interaction with protein, (B) hydrogen bonding interactions (C) hydrophobic interactions.

**Fig. 4:**
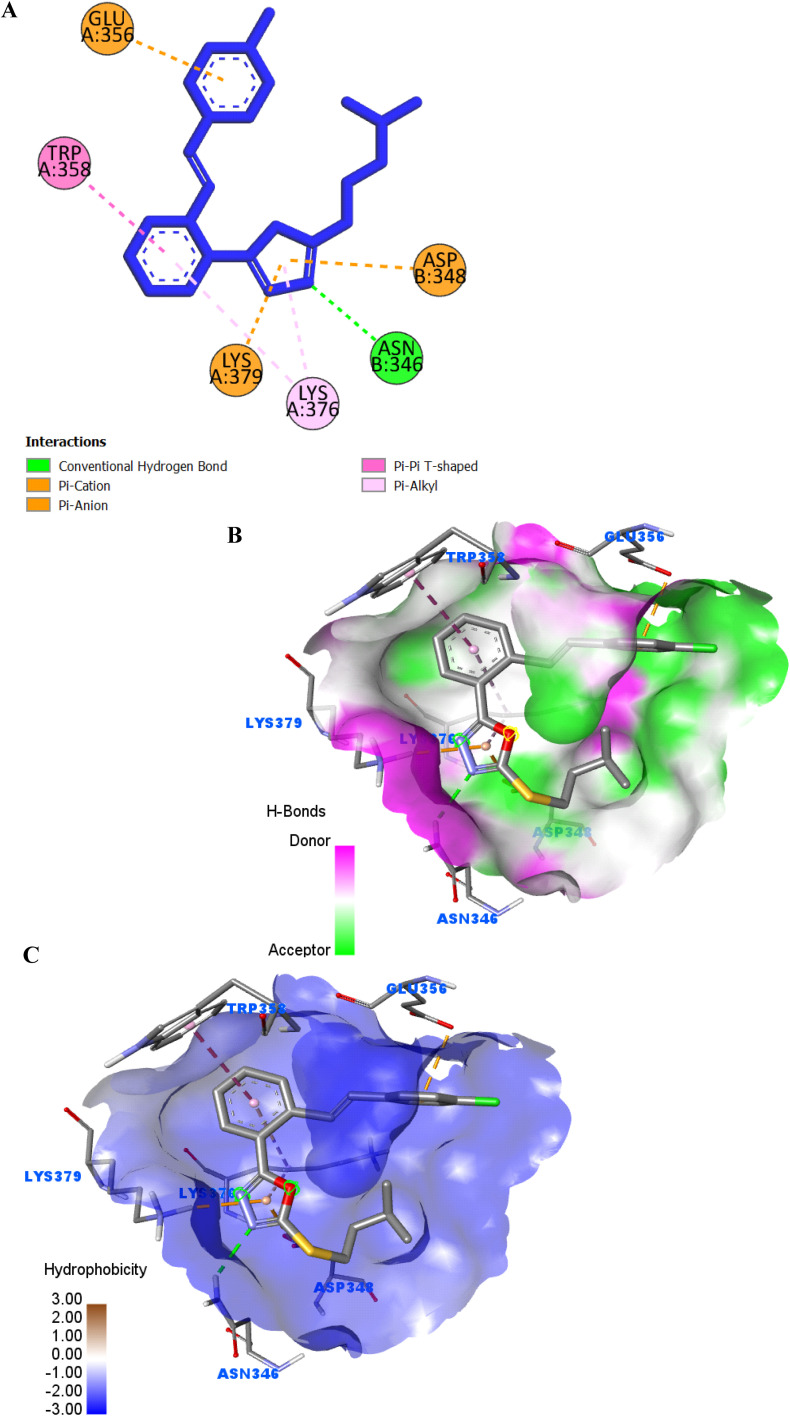
Docking studies of the synthesized compound 5b (A) 2D ligand interaction with protein, (B) hydrogen bonding interactions (C) hydrophobic interactions.

In accordance with the docking results, the oxadiazole derivative 5d exhibited a good fit of binding with the catalytic residues at the binding site of the protein, having good docking score *i.e.*, −10.0 kcal mol^−1^ as compared to the kojic and ascorbic acid, having −5.3 kcal mol^−1^ and −5.9 kcal mol^−1^ binding scores respectively (Table 2). It showed good affinities for the following amino acids of the binding pocket of enzyme: GLU-97, TYR-62, ILE-328, LEU-75, PRO-338, PRO-349. Compound 5d has one oxadiazole ring which exhibited strong hydrophilic interactions *i.e.*, π–donor hydrogen bond with TYR-62 amino acid of the protein. The halogen substituted aromatic ring displayed an electrostatic interaction which is π–anion interaction with GLU-97 amino acid. The chloro-group exhibited hydrophobic alkyl interaction with LEU-75 amino acid. Moreover, the two aromatic rings formed π–sigma and π–alkyl interactions with the ILE-328, PRO-338 and PRO-349 amino acids of the protein (Fig. 3). The compound 5b also displayed higher interaction energy (−8.5 kcal mol^−1^) as compared to the standards but lower than the compound 5d, validating the *in vitro* findings. The five membered heterocyclic ring exhibited several significant interactions including conventional hydrogen bonding interactions with residue ASN-346, π–cation interactions, π–anion interactions and π–alkyl interactions with residues LYS-379, ASP-348 and LYS-376 residues respectively. Moreover, the aromatic rings displayed several π interactions as halogen substituted aromatic ring formed π–anion interaction with GLU-356 residue and the other aromatic ring engage in π–π T-shaped binding with TRP-358 and LYS-376 residues (Fig. 4). Both the standards displayed strong hydrophilic interaction with binding pockets of protein as kojic acid exhibited strong hydrogen bonding *via* GLN-307, GLU-356 and THR-308 residues and ascorbic acid interacted with SER-380, TYR-58 and GLN-90 residues of protein. The distances of the bindings of ligands with the amino acid residues are elaborated in Table 3.

**Table 2:** Molecular docking scores of the compounds 5d and 5b along with standards

Sr. no.	Compounds	Docking score (kcal mol^−1^)
1	5d	−10.0
2	5b	−8.5
3	Kojic acid	−5.3
4	Ascorbic acid	−5.9

**Table 3:** Molecular docking interactions of the selected compounds 5d & 5b along with standards

Sr. no.	Compounds	Type of interactions	Amino acid residues involved in ligand–enzyme interactions	Distances (Å)
1	5d	π–Anion	GLU-97	4.511
		π–Donor hydrogen bond	TYR-62	3.25012
		π–Sigma	ILE-328	3.70719
		Alkyl	LEU-75	4.2881
		π–Alkyl	PRO-338	4.97602
			PRO-349	4.15998
2	5b	Conventional H-boding	ASN-346	2.37332
		π–Cation	LYS-379	2.65679
		π–Anion	GLU-356	3.44776
			ASP-348	3.97574
		π–π T-shaped	TRP-358	5.33213
		π–Alkyl	LYS-376	4.9203
			LYS-376	5.25125
3	Kojic acid	Hydrogen bonding	GLN-307	3.0289
			GLN-307	2.25317
			GLU-356	3.20052
			THR-308	2.92972
		π–π stacked	TRP-358	4.43245
4	Ascorbic acid	Hydrogen bonding	SER-380	2.80308
			TYR-58	2.87481
			GLN-90	2.36815
			SER380	2.88594
		C–H bond	LYS-93	3.52085
			ASN-310	3.43907

#### 3.3.2 Molecular dynamics simulations

Molecular dynamics (MD) simulation study is utilized to elucidate the atomic-level fluctuations, ligand–protein interactions and time-dependent structural dynamics of the ligand–protein complex. In order to evaluate the stability of docked poses generated by the docking analysis and to analyze binding characteristics of ligand and protein, the compound 5d was subjected to a 100 ns MD simulation analysis along with the reference drug *i.e.*, ascorbic acid for a comparative study against the tyrosinase target. The MD results were predicted using RMSD, RMSF and ligand–protein contact analysis.

##### 3.3.2.1 RMSD analysis of the tyrosinase inhibitors

**Fig. 5:**
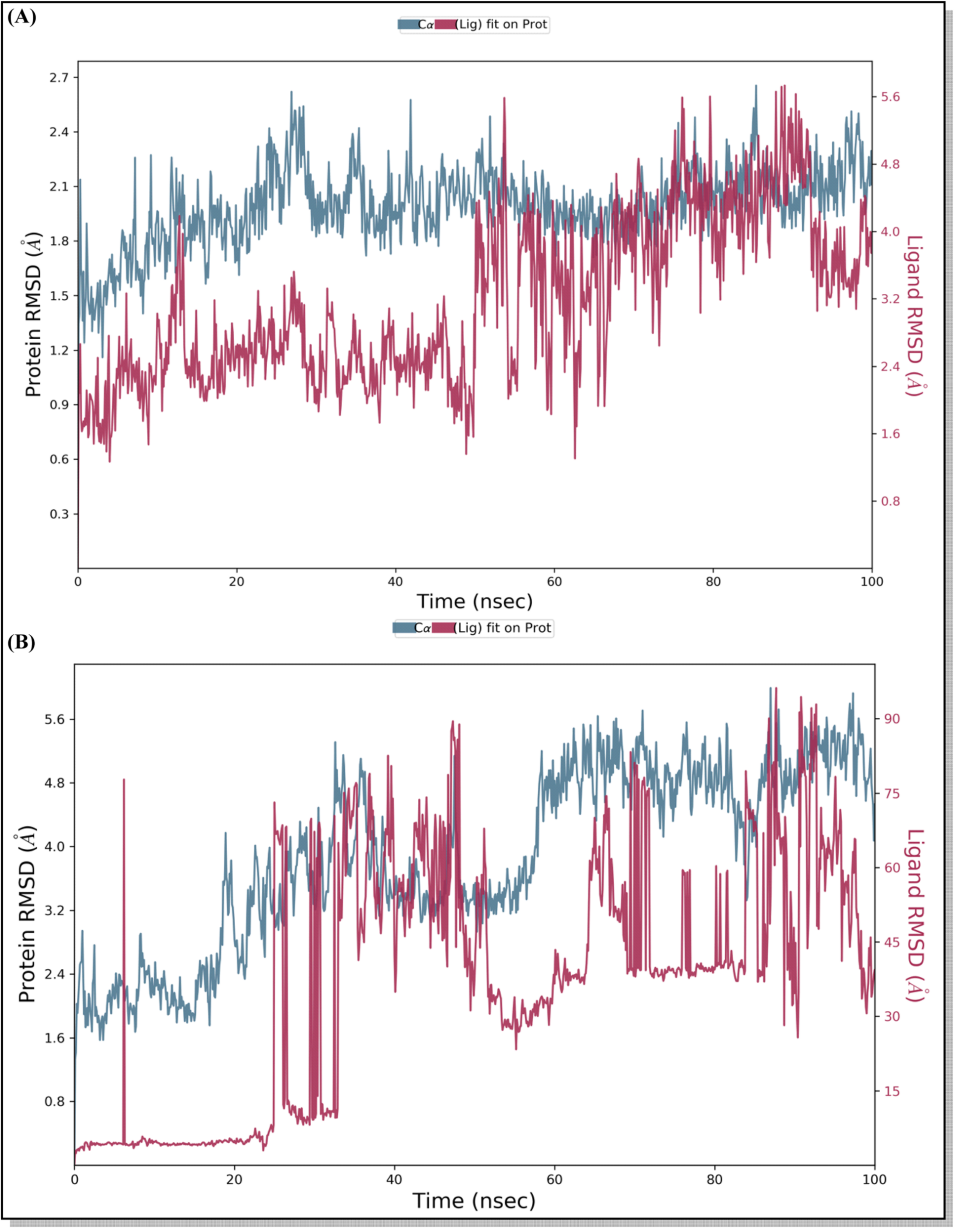
The RMSD of protein backbone structure (A) 5d and (B) ascorbic acid.

The protein Cα RMSD is a significant variable of MD simulation, employed to predict Cα deviation in a dynamic setting. It was observed that the 5d–protein complex showcased higher structural stability than reference–protein complex. The RMSD of 5d–protein complex fluctuated in the first phase of simulation up to 10 ns and then displayed a stable configuration until 50 ns. Within the range of 50–60 ns, the ligand showcased higher fluctuations, which were stabilized towards the end of simulation. The RMSD plot for 5d revealed that the protein backbone depicted fluctuations in the range of 1.2–2.6 Å while the ligand fluctuates over a range of 1.1–5.7 Å (Fig. 5A). The reference–protein complex exhibits higher fluctuation as compared to the 5d–protein complex and showed relatively stable configuration over a period of 35–50 ns simulation (Fig. 5B).

##### 3.3.2.2 RMSF analysis of the tyrosinase inhibitors

**Fig. 6:**
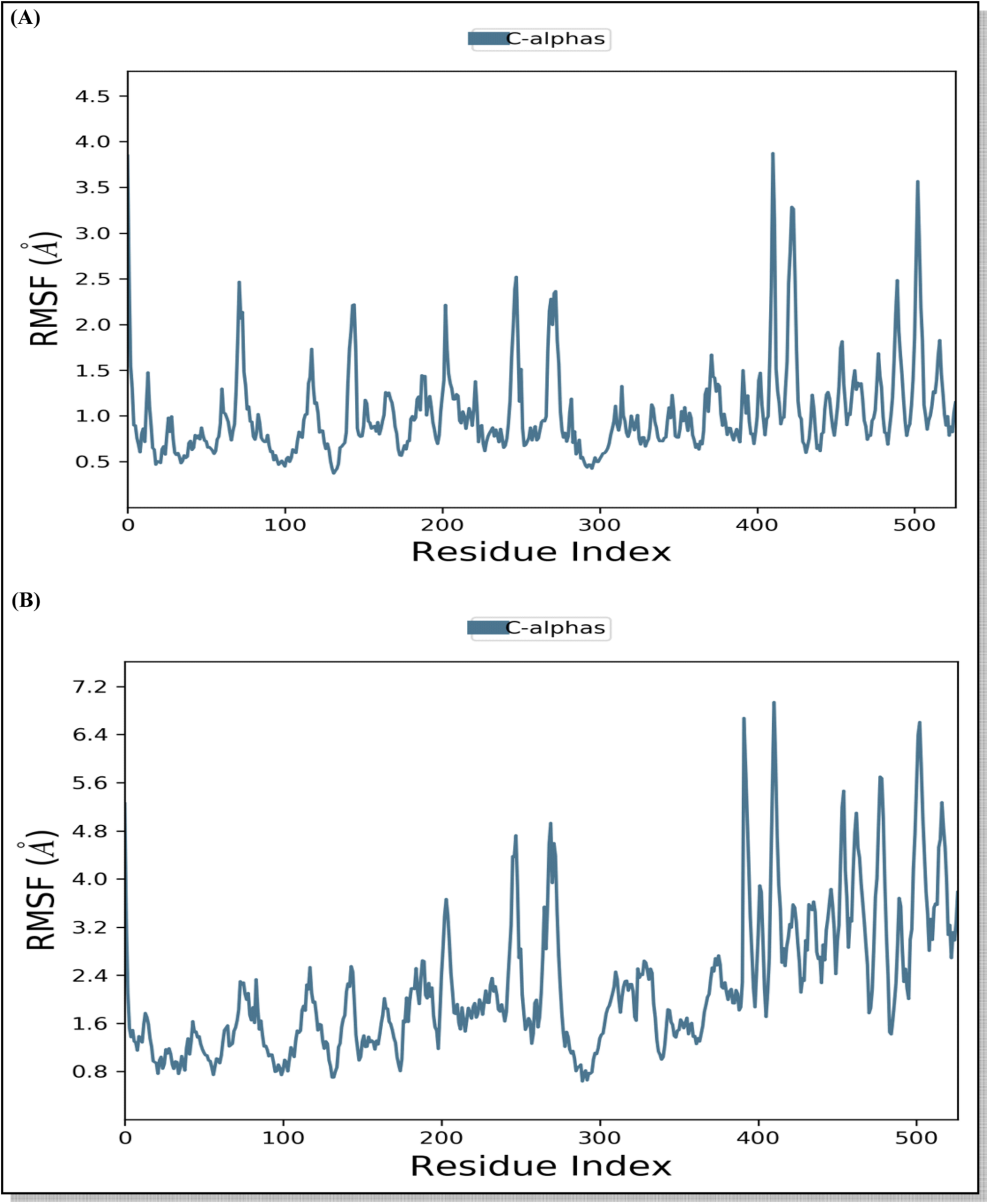
Protein RMSF plot (A) 5d and (B) ascorbic acid.

RMSF analysis is a measure of average fluctuations of atoms or residues, which were computed over a 100 ns simulation time. Higher peaks correspond to those amino acid residues which fluctuates the most during specified simulation period. MD studies revealed that the complex 5d–2y9x showcased higher fluctuations with amino acids THR-28, SER-126, PRO-46, GLY-47 and SER-35 with RMSF values within the range of 3.14–3.87 Å. Overall, the RMSF values for the whole protein falls within the range of 0.37–3.87 Å as depicted in Fig. 6A. The RMSF values for the ascorbic acid–protein complex were observed within the range of 0.69–6.93 Å. The amino acids which displayed higher fluctuations are THR-28, LEU-9 and ASP-10 (Fig. 6B).

##### 3.3.2.3 Protein–ligand contact analysis of tyrosinase inhibitors

**Fig. 7:**
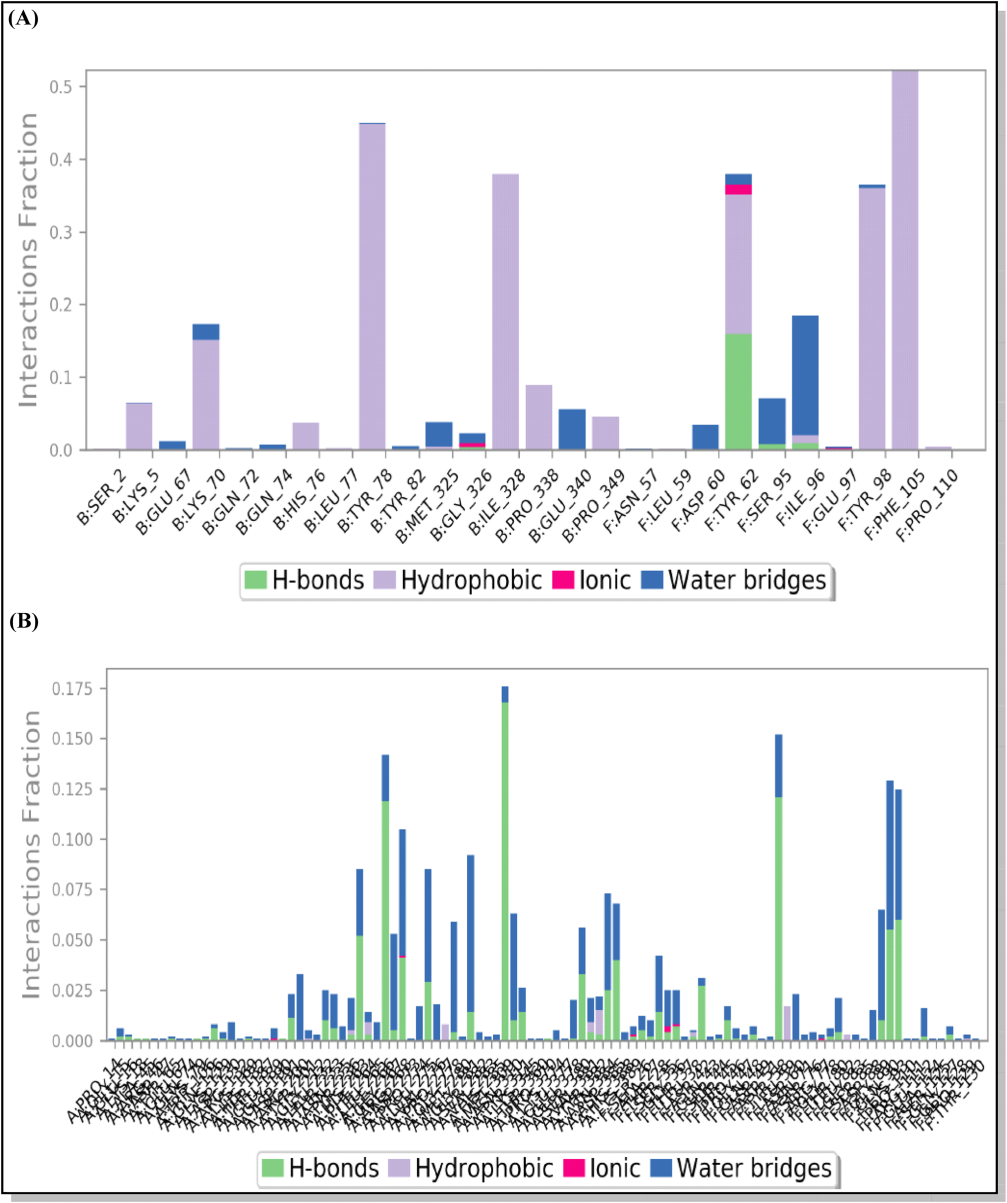
Plot (stacked bar charts) of protein–ligand contact analysis (A) 5d and (B) ascorbic acid.

Protein–ligand contact analysis is utilized to elucidate the key binding interactions shown by ligand atoms with protein residues throughout the simulation period. The 5d–2y9x complex displayed significant binding interaction with 26 amino acids of the protein depicted in Fig. 7. The ligand–protein contacts are classified into four types *i.e.*, H-bonding, hydrophobic, ionic interactions and water bridges. The ligand displayed strong hydrophobic interactions, less pronounced hydrogen bonding along with water mediated indirect hydrogen bonding and some extents of ionic interactions. The phenyl ring directly attached to the heterocyclic ring exhibit strong π–π stacking interactions with PHE-105. The other residues which displayed prominent hydrophobic interactions are SER-2, LYS-5, LYS-70, HIS-76, LEU-77, TYR-78, ILE-328, PRO-338, PRO-349 GLU-97, TYR-98, PHE-105 and PRO-110. Moreover, ligand–protein complex established H-bond interactions with the amino acids GLU-67, GLN-74, GLU-340 and ASP-60. Various residues displayed multiple interactions with the ligand atoms such as GLY-326 exhibit indirect H-bonding and ionic interactions. Similarly, TYR-62 showed direct and indirect H-bonding, hydrophobic and ionic interactions and SER-95 depicted water mediated H-bonding and direct H-bonding, whereas, ILE-96 exhibited H-bonding, water mediated H-bonding and some extent of hydrophobic interactions (Fig. 7A). The reference–protein complex exhibits strong H-bonding with the number of protein amino acid residues and displayed comparable results (Fig. 7B).

#### 3.3.3 DFT analysis

**Fig. 8:**
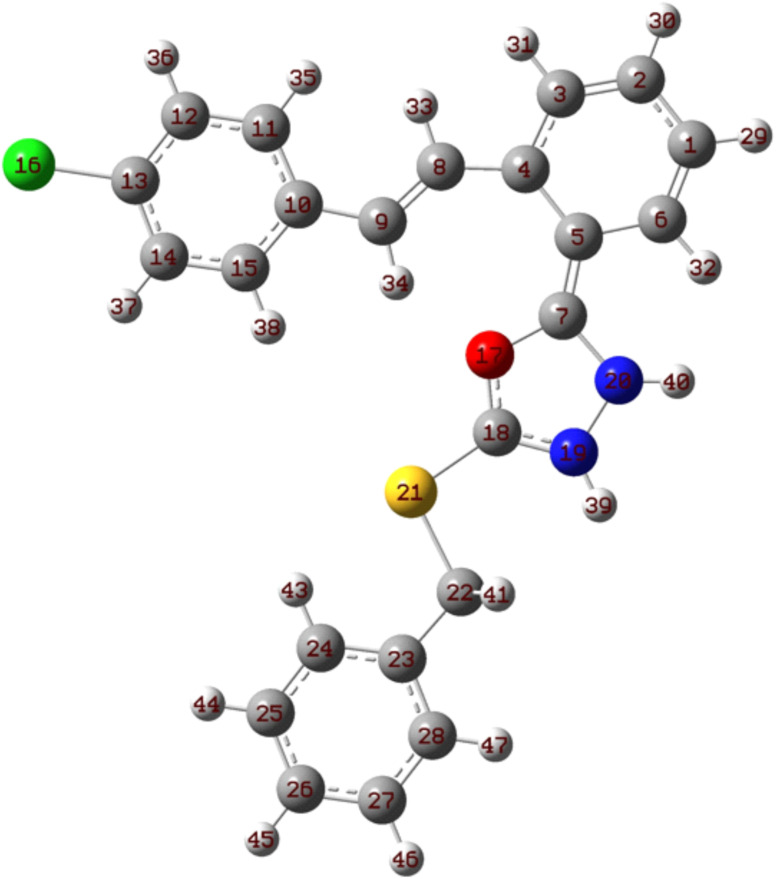
Optimized molecular structure of potent compound 5d.

The optimized structure and key electronic parameters (such as *E*_HOMO_ − *E*_LUMO_, molecular electrostatic potential, and Mulliken atomic charges *etc.*) of the compounds were analyzed employing B3LYP/6-31G basis set. The optimized structure, which refers to the most stable configuration of the compound under lowest energy state, of the compound 5d is given in Fig. 8.

##### 3.3.3.1 Frontier molecular orbital studies

**Fig. 9:**
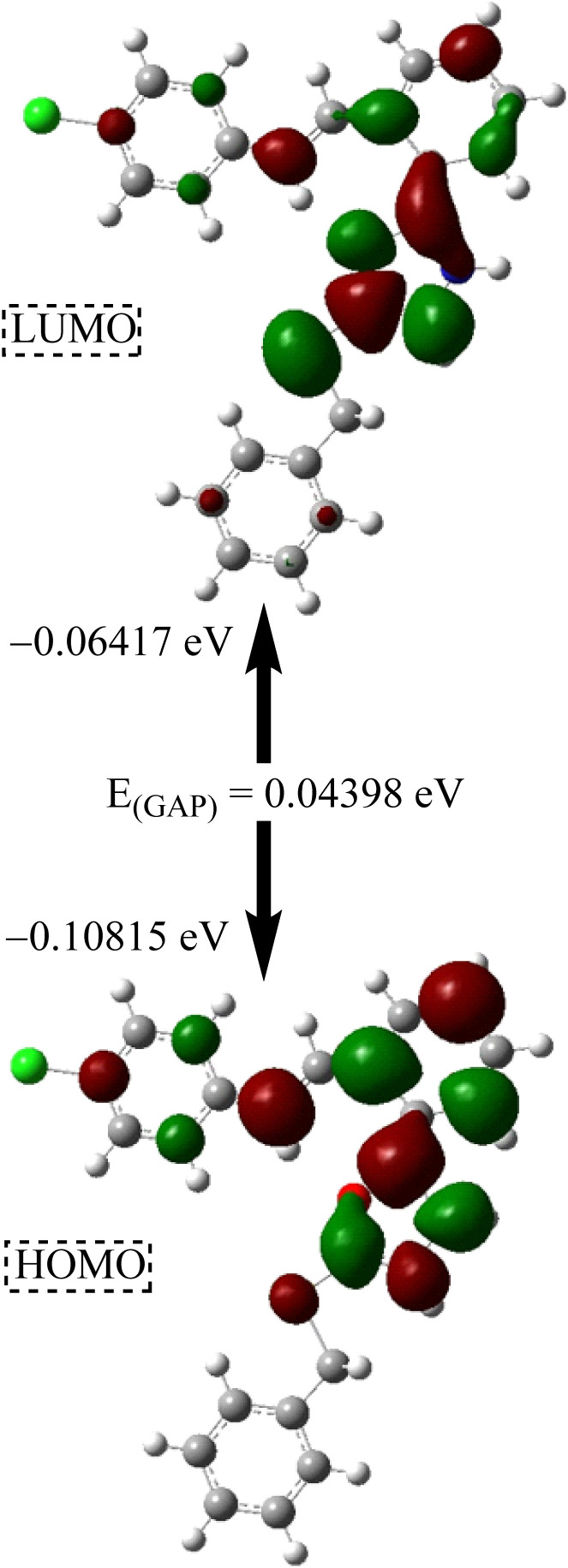
HOMO and LUMO molecular orbitals of compounds 5d.

The HOMO and LUMO molecular orbitals of potent compound 5d with calculated HOMO–LUMO energies and bond energies are depicted in Fig. 9. The reactivity and stability in terms of electron donating and accepting affinity were elucidated using HOMO and LUMO energies and the energy gap of the synthesized bioactive compound was calculated.^49^ The *E*_HOMO_ and *E*_LUMO_ values for compound 5d were determined to be −0.10815 eV and −0.06417 eV correspondingly with energy gap of 0.04398 eV. Global molecular descriptors are feature vectors which describe the overall characteristics of the compounds. These include ionization energy, electron affinity, chemical potential, electronegativity, electrostatic index, chemical hardness and softness.^50^ The key characteristics of each compound were calculated and depicted in the table (Table 4).

**Table 4:** Calculated global descriptors of the compound 5d^a^

Electronic parameters (eV)	5d
*E*_(HOMO)_	−0.10815
*E*_(LUMO)_	−0.06417
IE	0.10815
EA	0.06417
*X*	0.08616
*µ*	−0.08616
*ω*	0.1688
*η*	0.02199
*S*	45.475

a IE = ionization energy, EA = electron affinity, *X* = electronegativity, *µ* = chemical potential, *ω* = electrophilicity index, *η* = chemical hardness, *S* = chemical softness.

##### 3.3.3.2 Molecular electrostatic potential analysis of the compounds (MEP)

**Fig. 10:**
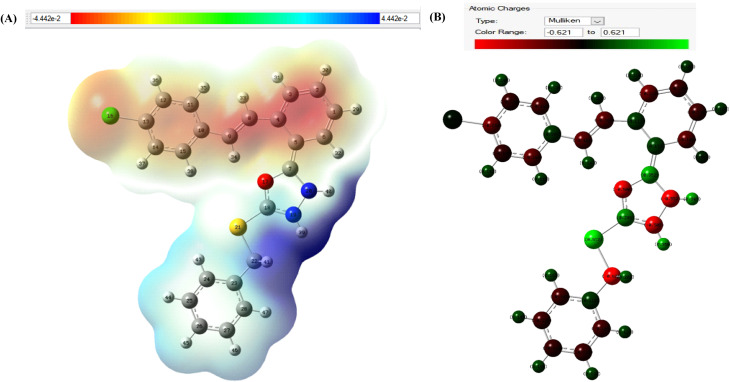
(A) MEP map and (B) calculated Mulliken atomic charges of compound 5d.

In order to illustrate molecule's charge distribution, reactivity, molecular properties of the synthesized compound, the molecular surface electrostatic potential was documented. It is also used to visualize the relative polarity and charge density of the compounds.^51^ The MEP analysis for the potent compound is depicted in Fig. 10A. The map's color scale ranges within −4.443 × 10^−2^ and 4.443 × 10^−2^. The color distribution over the compound structure illustrated the charge distribution as negative region showing red and yellow colors correlated with nucleophilic behavior and positive regions represented by blue color are characterized by electrophilic behavior of the compound of 5d.

##### 3.3.3.3 Mulliken atomic charges

To measure the partial atomic charges, dipole moment, refractivity as well as to indicate adsorptive sites for the compounds, Mulliken atomic charges analysis was established.^52^ The Mulliken atomic charges were measured at the 6-31G basis set. The map's color scale ranges within −0.621 and 0.621. From the calculated analysis, it was found that almost all the C-atoms in the 5d exhibited negative charges except for the carbon linked to the heterocycle and the carbon atom present at the positions where separate phenyl rings were linked. However, the hydrogen atoms linked with these C-atoms and the nitrogen atoms in compound 5d showed positive atomic charges. The sulfur group depicted highly electropositive behavior along with the carbon atoms present in the heterocycle. Both the nitrogen and the oxygen present in the heterocycle displayed prominent positive Mulliken charges (Fig. 10B).

#### 3.3.4 ADMET and other physiochemical properties

ADMET (absorption, distribution, metabolism, excretion, and toxicity) has profound influence in drug development as it predicts pharmacokinetic characteristics of drugs. ADMET analysis facilitates the scientists to identify potential leads in pharmaceutics. In this perspective, all synthesized derivatives 4a–b and 5a–f were investigated to predict their ADMET and other physiochemical properties utilizing ADMETlab 3.0. By using this software, the physiochemical and medicinal properties such as molecular weight (MW), topological polar surface area (TPSA), number of hydrogen bond donors (*n*_HD_), number of hydrogen bond acceptors (*n*_HA_), partition coefficient (log *P*), number of rotatable bonds (*n*_Rot_) of the synthesized analogues were determined (Table 5). Lipinski's rule is one of the tools to predict drug-likeness of analyzed compounds and in accordance to this rule, the drug must possess the following physiochemical features (i) MW must be equal or less than 500 Da (ii) *n*_HA_ must be less than 10 (iii) *n*_HD_ equal or less than 5 (iv) log *P* should be lower than 5. The compounds (5a–5f) exhibit log *P* values exceeding 5 (ranges 5.25–7.82, Table 5), indicating elevated lipophilicity in relation to Lipinski's Rule of Five. While increased lipophilicity may favor membrane permeability and passive diffusion, it is typically associated with reduced aqueous solubility. Accordingly, the solubility profile of these derivatives represents a notable physicochemical limitation that should be addressed in future structural optimization.

**Table 5:** Predicted physiochemical and medicinal profile of compound 4a–5f along with the standards^a^

Sr. no.	Compounds	MW	Volume	*n*_HA_	*n*_HD_	Log *P*	*n*_Rot_	TPSA (Å)	Lipinski rule
1	4a	280.07	285.187	3.0	0.0	3.901	3.0	38.92	Acceptable
2	4b	314.03	300.399	3.0	0.0	4.672	3.0	38.92	Acceptable
3	5a	350.15	371.667	3.0	0.0	5.723	7.0	38.92	Acceptable
4	5b	384.11	386.878	3.0	0.0	5.972	7.0	38.92	Acceptable
5	5c	370.11	389.794	3.0	0.0	5.255	6.0	38.92	Acceptable
6	5d	404.08	405.005	3.0	0.0	5.875	6.0	38.92	Acceptable
7	5e	392.19	423.555	3.0	0.0	7.392	11.0	38.92	Acceptable
8	5f	426.15	438.766	3.0	0.0	7.82	11.0	38.92	Acceptable
9	Kojic acid	142.03	131.027	4.0	2.0	−0.565	1.0	70.67	Acceptable
10	Ascorbic acid	176.03	151.244	6.0	4.0	−1.707	2.0	107.22	Acceptable

a MW: molecular weight, *n*_HA_: number of hydrogen acceptors, *n*_HD_: number of hydrogen donors, log *P*: water partition coefficient, *n*_Rot_: number of rotatable bonds, TPSA: topological polar surface area.

Furthermore, the other ADMET attributes such as absorption, distribution, metabolism, excretion and toxicity profiles were also analyzed for the detailed insights of the synthesized compounds 4a–b and 5a–f, with kojic and ascorbic acid as standards (Table 6).

**Table 6:** Predicted ADME profile of the compounds 4a–b and 5a–f along with the standards^a^

Sr. no.	Compounds	Caco-2 permeability	MDCK permeability	PPB	BBB	CYP1A2 inhibitor	CYP2C19 inhibitor	HLM-stability	CL_plasma_
1	4a	−4.878	−4.705	98.665	0.003	1.0	0.829	0.244	7.544
2	4b	−4.856	−4.708	99.238	0.008	1.0	0.981	0.051	7.205
3	5a	−4.983	−4.693	98.983	0.543	1.0	1.0	0.956	7.735
4	5b	−5.021	−4.676	99.03	0.692	1.0	1.0	0.805	7.526
5	5c	−4.947	−4.695	97.84	0.374	1.0	1.0	0.193	5.98
6	5d	−4.938	−4.683	98.369	0.531	1.0	1.0	0.044	5.943
7	5e	−5.075	−4.682	99.589	0.372	1.0	1.0	0.564	5.737
8	5f	−5.043	−4.686	99.462	0.528	1.0	1.0	0.198	5.516
9	Kojic acid	−4.86	−4.613	23.332	0.055	0.068	0.001	0.013	11.409
10	Ascorbic acid	−6.009	−4.808	39.176	0.685	0.083	0.0	0.247	5.51

a PPB: plasma protein binding, BBB: blood–brain barrier.

The high predicted skin-sensitization probabilities (0.972–0.995) in Table 7 highlight a potential safety concern for topical use, indicating that these compounds should be considered as lead structures requiring further validation through *in vitro* cytotoxicity, skin irritation and sensitizations studies essential to validate their further development. The computational results should be interpreted as supportive rather than definitive of the experimental findings.

**Table 7:** Predicted toxicokinetic analyses of the compound 4a–5f along with the standards^a^

Sr. no.	Compounds	FDAMDD	Carcinogenicity	Skin-sensitization	Eye-corrosion	Eye-irritation	Ototoxicity	Human hepato-toxicity
1	4a	0.177	0.142	0.994	0.012	0.982	0.104	0.951
2	4b	0.17	0.127	0.995	0.007	0.949	0.156	0.951
3	5a	0.4	0.547	0.982	0.065	0.966	0.333	0.798
4	5b	0.394	0.518	0.985	0.037	0.907	0.442	0.798
5	5c	0.52	0.333	0.972	0.002	0.911	0.21	0.861
6	5d	0.513	0.308	0.977	0.001	0.78	0.297	0.862
7	5e	0.31	0.408	0.989	0.094	0.979	0.27	0.695
8	5f	0.304	0.381	0.991	0.055	0.942	0.37	0.696
9	Kojic acid	0.546	0.74	0.323	0.613	0.989	0.156	0.399
10	Ascorbic acid	0.029	0.314	0.967	0.103	0.979	0.67	0.753

a FDAMDD: FDA maximum daily dose.

## 4. Conclusion

Oxadiazole-based scaffolds continue to demonstrate significant potential in medicinal chemistry. In the present study, a series of *E*-stilbene-bearing oxadiazole derivatives was successfully designed, synthesized, and evaluated for anti-tyrosinase activity. Most compounds exhibited notable inhibitory effects compared with standard inhibitors, with compounds 5b and 5d emerging as the most active derivatives. To further substantiate the experimental findings, comprehensive *in silico* investigations were conducted, including molecular docking and MD simulations, which confirmed the strong binding affinity and stability of the active compounds within the enzyme binding site. Additionally, the most potent derivative, 5d was explored through density functional theory (DFT) studies to determine their electronic properties. Parameters such as frontier molecular orbital (HOMO–LUMO) energies, molecular electrostatic potential (MEP) distributions, and Mulliken atomic charge analysis were evaluated to elucidate the reactivity patterns, charge distribution, and overall stability of the compound. All the synthesized derivatives were also investigated to predict their ADMET properties and met the acceptable crieteria of Lipinski rule of five, however, compounds 5a–f exhibited elevated lipophilicity (log *P* > 5), exceeding the recommended threshold and potentially compromising aqueous solubility. Despite that, due to their promising inhibitory profiles, these derivatives should be regarded as lead compounds for further optimization. The study demonstrated an efficient synthetic strategy and encouraging biological activity; however, the translational potential of these compounds requires careful consideration of their high lipophilicity and the predicted skin-sensitization risk. Addressing these limitations through structural refinement and experimental validation will further advance their potential toward clinical or cosmetic applications.

## Author contributions

Aasia Javed: conceptualization, methodology, investigation, validation, writing – original draft. Zulfiqar Ali Khan: conceptualization, methodology, investigation, validation, supervision, project administration. Ameer Fawad Zahoor: conceptualization, methodology, investigation, validation, supervision, visualization, writing – review & editing.

## Conflicts of interest

There are no conflicts to declare.

## Supplementary Material

RA-OLF-D6RA03363F-s001

## Data Availability

All data is contained in the manuscript and supplementary information (SI) file. Supplementary information is available. See DOI: https://doi.org/10.1039/d6ra03363f.

## References

[cit1] Deri B., Kanteev M., Goldfeder M., Lecina D., Guallar V., Adir N., Fishman A. (2016). The unravelling of the complex pattern of tyrosinase inhibition. Sci. Rep..

[cit2] Zolghadri S., Bahrami A., Hassan Khan M. T., Muñoz-Muñoz J., García-Molina F., García-Cánovas F., Saboury A. A. (2019). A comprehensive review on tyrosinase inhibitors. J. Enzyme Inhib. Med. Chem..

[cit3] Chang T. S. (2009). An updated review of tyrosinase inhibitors. Int. J. Mol. Sci..

[cit4] Solomon E. I., Sundaram U. M., Machonkin T. E. (1996). Multicopper oxidases and oxygenases. Chem. Rev..

[cit5] Kim Y. M., Yun J., Lee C. K., Lee H., Min K. R., Kim Y. (2002). Oxyresveratrol and hydroxystilbene compounds: inhibitory effect on tyrosinase and mechanism of action. J. Biol. Chem..

[cit6] Mayer A. M. (2006). Polyphenol oxidases in plants and fungi: going places? A review. Phytochemistry.

[cit7] Jaenicke E., Decker H. (2003). Tyrosinases from crustaceans form hexamers. Biochem. J..

[cit8] Nagatsu T., Nakashima A., Watanabe H., Ito S., Wakamatsu K. (2022). Neuromelanin in Parkinson's disease: tyrosine hydroxylase and tyrosinase. Int. J. Mol. Sci..

[cit9] Chen H.-Y., Yeh Y.-C. (2020). Detection of tyrosine and monitoring tyrosinase activity using an enzyme cascade-triggered colorimetric reaction. RSC Adv..

[cit10] Rodríguez-López J. N., Tudela J., Varón R., García-Cánovas F. (1991). Kinetic study on the effect of pH on the melanin biosynthesis pathway. Biochim. Biophys. Acta.

[cit11] Cai H., Wen H., Li J., Lu L., Zhao W., Jiang X., Bai R. (2024). Small-molecule agents for treating skin diseases. Eur. J. Med. Chem..

[cit12] Frenk E. , Melasma: clinical and epidemiological features, in Melasma: New Approaches to Treatment, Martin Dunitz, London, 1995, pp. 9–15

[cit13] Nagatsu T., Nakashima A., Watanabe H., Ito S., Wakamatsu K., Zucca F. A., Zecca L., Youdim M., Wulf M., Riederer P., Dijkstra J. M. (2023). The role of tyrosine hydroxylase as a key player in neuromelanin synthesis and the association of neuromelanin with Parkinson's disease. J. Neural Transm..

[cit14] Mayer A. M. (1986). Polyphenol oxidases in plants-recent progress. Phytochemistry.

[cit15] Whitaker J. R. , Polyphenol oxidase, in Food Enzymes: Structure and Mechanism, ed. D. W. S. Wong, Chapman and Hall, New York, 1995, pp. 271–307.

[cit16] Kim Y. J., Uyama H. (2005). Tyrosinase inhibitors from natural and synthetic sources: structure, inhibition mechanism and perspective for the future. Cell. Mol. Life Sci..

[cit17] Marieshwari B. N., Bhuvaragavan S., Sruthi K., Mullainadhan P., Janarthanan S. (2023). Insect phenoloxidase and its diverse roles: melanogenesis and beyond. J. Comp. Physiol. B.

[cit18] Carradori S., Melfi F., Rešetar J., Şimşek R. (2024). Tyrosinase enzyme and its inhibitors: an update of the literature. Metalloenzymes.

[cit19] Briganti S., Camera E., Picardo M. (2003). Chemical and instrumental approaches to treat hyperpigmentation. Pigm. Cell Res..

[cit20] Momtaz S., Lall N., Basson A. (2008). Inhibitory activities of mushroom tyrosine and DOPA oxidation by plant extracts. S. Afr. J. Bot..

[cit21] Khan M. T. H. , in Bioactive Heterocycles III, Springer, 2007

[cit22] Ullah S., Son S., Yun H. Y., Kim D. H., Chun P., Moon H. R. (2016). Tyrosinase inhibitors: a patent review (2011-2015). Expert Opin. Ther. Pat..

[cit23] De B., Adhikari I., Nandy A., Saha A., Goswami B. B. (2018). In silico modelling of azole derivatives with tyrosinase inhibition ability: application of the models for activity prediction of new compounds. Comput. Biol. Chem..

[cit24] Chandarajoti K., Kara J., Suwanhom P., Nualnoi T., Puripattanavong J., Lee V. S., Tipmanee V., Lomlim L. (2024). Synthesis and evaluation of coumarin derivatives on antioxidative, tyrosinase inhibitory activities, melanogenesis, and in silico investigations. Sci. Rep..

[cit25] Vanjare B. D., Choi N. G., Mahajan P. G., Raza H., Hassan M., Han Y., Yu S.-M., Kim S. J., Seo S.-Y., Lee K. H. (2021). Novel 1, 3, 4-oxadiazole compounds inhibit the tyrosinase and melanin level: synthesis, in-vitro, and in-silico studies. Bioorg. Med. Chem..

[cit26] Khan A., Elhenawy A. A., Rehman M. U., Alam M., Alam A., Rehman N. U., Ibrahim M. (2024). Synthesis of novel 2-mercapto-1, 3, 4-oxadiazole derivatives as potent urease inhibitors: in vitro and in silico investigations. J. Mol. Struct..

[cit27] Begum F., Yousaf M., Iqbal S., Ullah N., Hussain A., Khan M., Khalid A., Algarni A. S., Addalla A. N., Khan A, Lodhi M. A., Al-Harrasi A. (2023). Inhibition of acetylcholinesterase with novel 1, 3, 4, oxadiazole derivatives: a kinetic, in silico, and in vitro approach. ACS Omega.

[cit28] Bianco G., Meleddu R., Distinto S., Cottiglia F., Gaspari M., Melis C., Corona A., Angius R., Angeli A., Taverna D., Alcaro S., Leitans J., Kazaks A., Tars K., Supuran C. T., Maccioni E. (2017). *N*-Acylbenzenesulfonamide dihydro-1, 3, 4-oxadiazole hybrids: seeking selectivity toward carbonic anhydrase isoforms. ACS Med. Chem. Lett..

[cit29] Vaidya A., Pathak D., Shah K. (2021). 1, 3, 4‐oxadiazole and its derivatives: a review on recent progress in anticancer activities. Chem. Biol. Drug Des..

[cit30] Mishra A. K., Kumar A., Sahu J. K. (2020). Recent advancements in biological activities of oxadiazole and their derivatives: a review. Lett. Org. Chem..

[cit31] Siwach A., Verma P. K. (2020). Therapeutic potential of oxadiazole or furadiazole containing compounds. BMC Chem..

[cit32] Olivares C., Solano F. (2009). New insights into the active site structure and catalytic mechanism of tyrosinase and its related proteins. Pigm. Cell Melanoma Res..

[cit33] Khan Z. A., Iqbal A., Shahzad S. A. (2017). Synthetic approaches toward stilbenes and their related structures. Mol. Diversity.

[cit34] Bernard P., Berthon J.-Y. (2000). Resveratrol: an original mechanism on tyrosinase inhibition. Int. J. Cosmet. Sci..

[cit35] Satooka H., Kubo I. (2012). Resveratrol as a kcat type inhibitor for tyrosinase: potentiated melanogenesis inhibitor. Bioorg. Med. Chem..

[cit36] Morais T. S. (2024). Recent advances in the development of hybrid drugs. Pharmaceutics.

[cit37] Javed A., Khan Z. A., Khushal A., Khan S., Farooq U., Shahzad S. A. (2026). E‐Stilbene‐Based Hydrazide‐1, 3, 4‐Oxadiazole Hybrids as Potential Anticancer Dual Carbonic Anhydrase‐I and Thymidine Phosphorylase Inhibitors: In Vitro and In Silico Studies. ChemistrySelect.

[cit38] Ellman G. L., Courtney K. D., Andres Jr V., Featherstone R. M. (1961). A new and rapid colorimetric determination of acetylcholinesterase activity. Biochem. Pharmacol..

[cit39] Bagheri A., Moradi S., Iraji A., Mahdavi M. (2024). Structure-based development of 3, 5-dihydroxybenzoyl-hydrazineylidene as tyrosinase inhibitor; in vitro and in silico study. Sci. Rep..

[cit40] Sripadung P., Rajchakom C., Nunthaboot N., Jiang X., Sungthong B. (2025). Computational and Experimental Insights into Tyrosinase and Antioxidant Activities of Resveratrol and Its Derivatives: Molecular Docking, Molecular Dynamics Simulation, DFT Calculation, and In Vitro Evaluation. Int. J. Mol. Sci..

[cit41] Moukhliss Y., Koubi Y., Alaqarbeh M., Alsakhen N., Hamzeh S., Maghat H., Sbai A., Bouachrine M., Lakhlifi T. (2022). A study of drug candidates derived from pleconaril for inhibiting coxsackievirus B3 (Cvb3) by ADMET, molecular docking, molecular dynamics and retrosynthesis. New J. Chem..

[cit42] Agda F., Nebbach D., Abram T., Bouachrine M., Taleb M. (2020). Photophysical properties of electroluminescent molecules based on thiophene and oxadiazole. Results Chem..

[cit43] Sadeghian S., Zare F., Khoshneviszadeh M., Hafshejani A. F., Salahshour F., Khodabakhshloo A., Saghaie L., Goshtasbi G., Sarikhani Z., Poustforoosh A., Sabet R., Sadeghpour H. (2024). Synthesis, biological evaluation, molecular docking, MD simulation and DFT analysis of new 3-hydroxypyridine-4-one derivatives as anti-tyrosinase and antioxidant agents. Heliyon.

[cit44] Fu L., Shi S., Yi J., Wang N., He Y., Wu Z., Peng J., Deng Y., Wang W., Wu C., Lyu A., Zeng X., Zhao W., Hou T., Cao D. (2024). ADMETlab 3.0: an updated comprehensive online ADMET prediction platform enhanced with broader coverage, improved performance, API functionality and decision support. Nucleic Acids Res..

[cit45] Iqbal A., Khan Z. A., Shahzad S. A., Khan S. A., Naqvi S. A. R., Bari A., Amjad H., Umar M. I. (2019). Synthesis, modeling studies and evaluation of E-stilbene hydrazides as potent anticancer agents. J. Mol. Struct..

[cit46] Beletskaya I. P., Cheprakov A. V. (2000). The Heck reaction as a sharpening stone of palladium catalysis. Chem. Rev..

[cit47] Irfan A., Zahoor A. F., Kamal S., Hassan M., Kloczkowski A. (2022). Ultrasonic-assisted synthesis of benzofuran appended oxadiazole molecules as tyrosinase inhibitors: mechanistic approach through enzyme inhibition, molecular docking, chemoinformatics, ADMET and drug-likeness studies. Int. J. Mol. Sci..

[cit48] Mushtaq A., Zahoor A. F., Kamal S., Mojzych M., Saif M. J., Bhat M. A. (2025). 7-Methoxybenzofuran-triazole tethered N-phenylacetamides as a promising class of tyrosinase inhibitors: synthesis, biological evaluation and computational analyses against fungal and human tyrosinases. RSC Adv..

[cit49] Siddique S. A., Arshad M., Naveed S., Mehboob M. Y., Adnan M., Hussain R., Ali B., Bilal M., Siddique A., Liu X. (2021). Efficient tuning of zinc phthalocyanine-based dyes for dye-sensitized solar cells: a detailed DFT study. RSC Adv..

[cit50] Miar M., Shiroudi A., Pourshamsian K., Oliaey A. R., Hatamjafari F. (2021). Theoretical investigations on the HOMO–LUMO gap and global reactivity descriptor studies, natural bond orbital, and nucleus-independent chemical shifts analyses of 3-phenylbenzo [d] thiazole-2 (3 H)-imine and its para-substituted derivatives: solvent and substituent effects. J. Chem. Res..

[cit51] Kaddouri Y., Bouchal B., Abrigach F., El Kodadi M., Bellaoui M., Touzani R. (2021). Synthesis, Molecular docking, MEP and SAR Analysis, ADME‐Tox predictions, and antimicrobial evaluation of novel mono‐and tetra‐alkylated pyrazole and triazole ligands. J. Chem..

[cit52] Qadr H. M., Mamand D. M. (2021). Molecular structure and density functional theory investigation corrosion inhibitors of some oxadiazoles. J. Bio- Tribo-Corros..

