# Supplementary material for: Design and computational evaluation of E-stilbene-bearing 1,3,4-oxadiazole derivatives as potential tyrosinase inhibitors using DFT, molecular docking and ADMET studies
Source: RSC Adv. 2026 Jul 7;16(36):38364–83. doi: 10.1039/d6ra03363f (PMC13338927; doi:10.1039/d6ra03363f)
Supplement: Supplementary file 1 [file RA-016-D6RA03363F-s001.pdf]

Supplementary Information (SI) for RSC Advances.
This journal is © The Royal Society of Chemistry 2026

Supporting Information Spectra

Design and Computational evaluation of E-stilbene-based 1,3,4-oxadiazole derivatives as potential tyrosinase inhibitors using DFT, molecular docking and ADMET studies

Fig. S1 FT-IR spectrum (KBr) of compound **5a**.

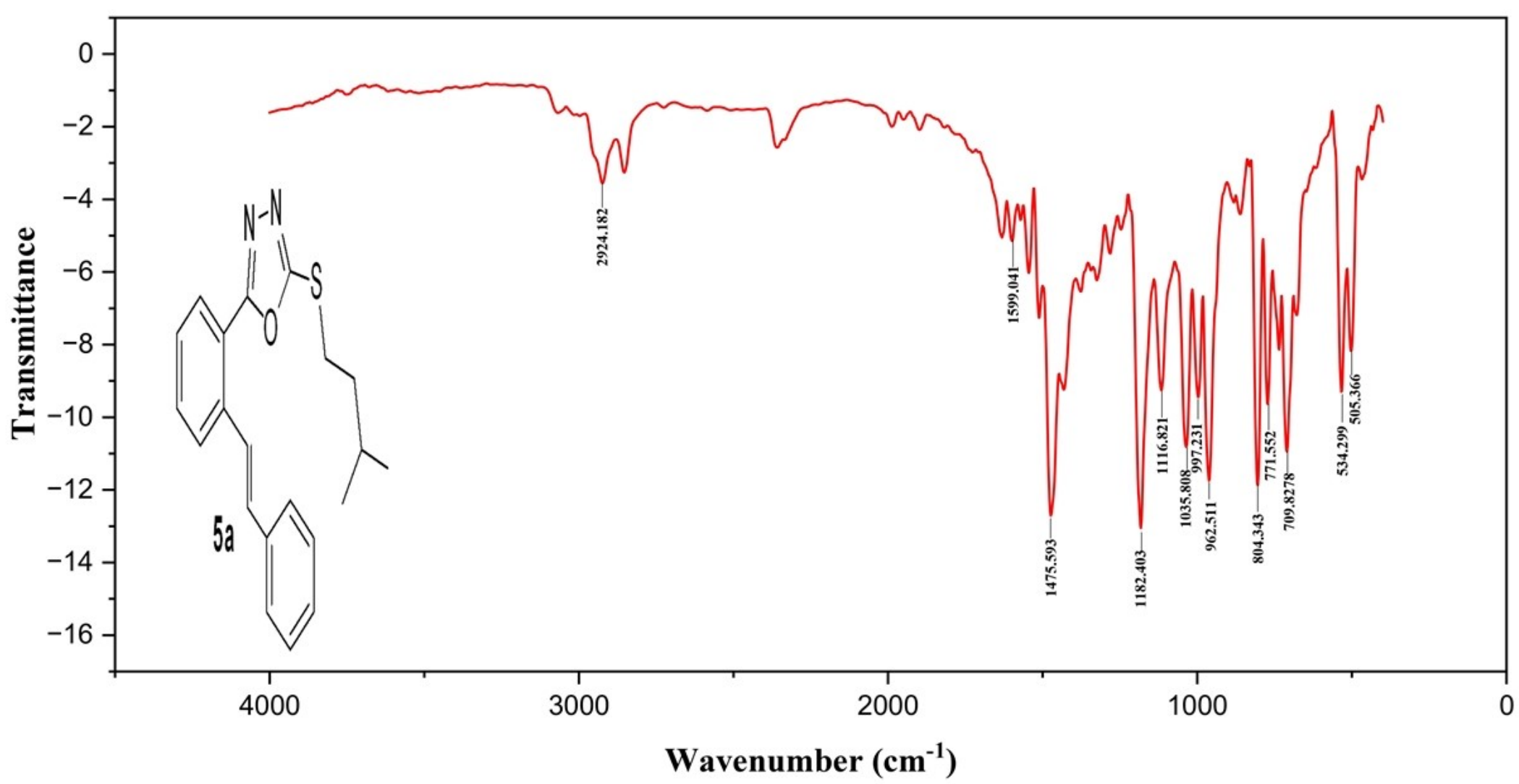

Fig. S2 ^{1}H NMR spectrum (400 MHz, $CDCl_3$) of compound **5a**.

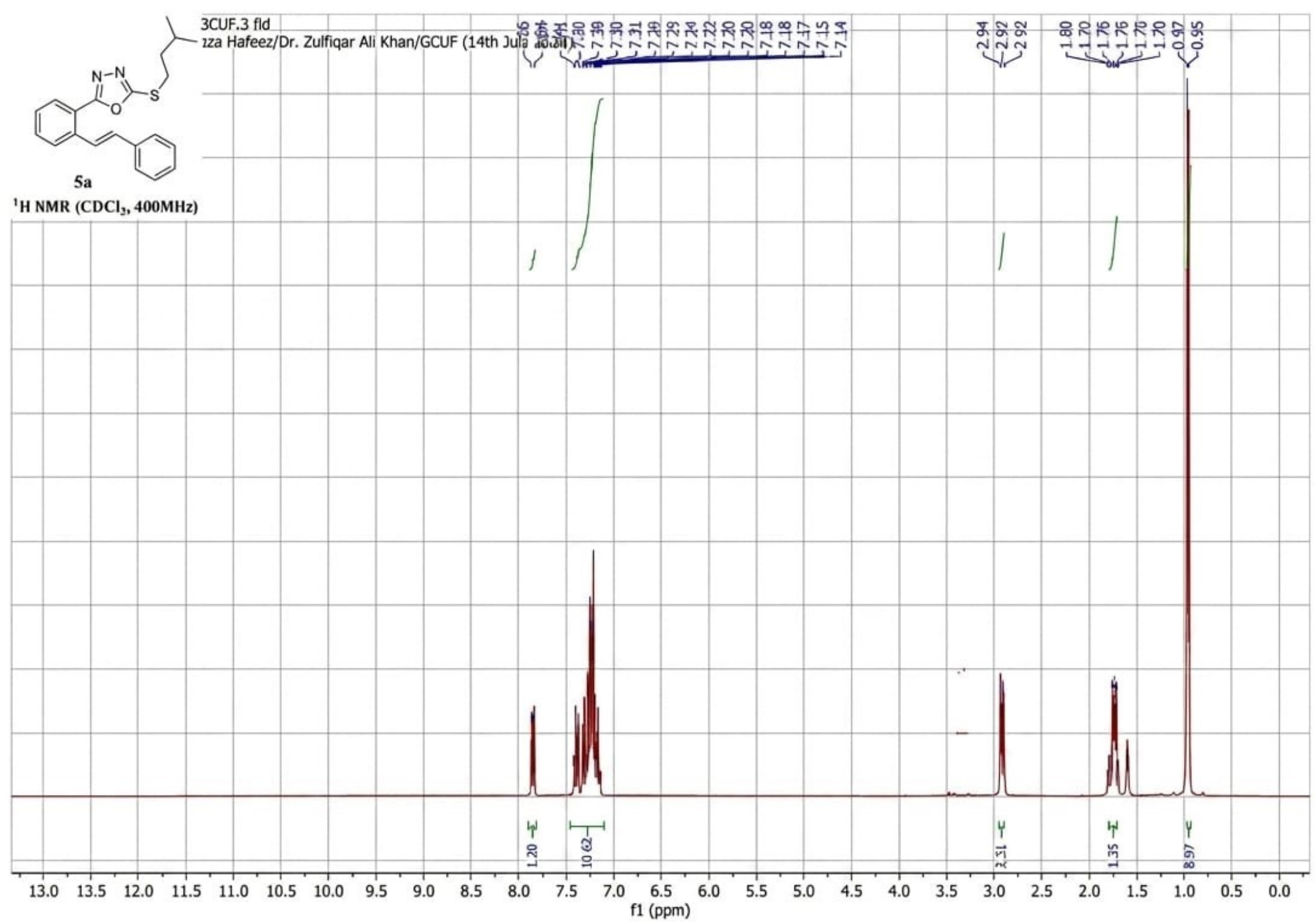

Fig. S3 ^{13}C NMR spectrum (100 MHz, $CDCl_3$) of compound **5a**.

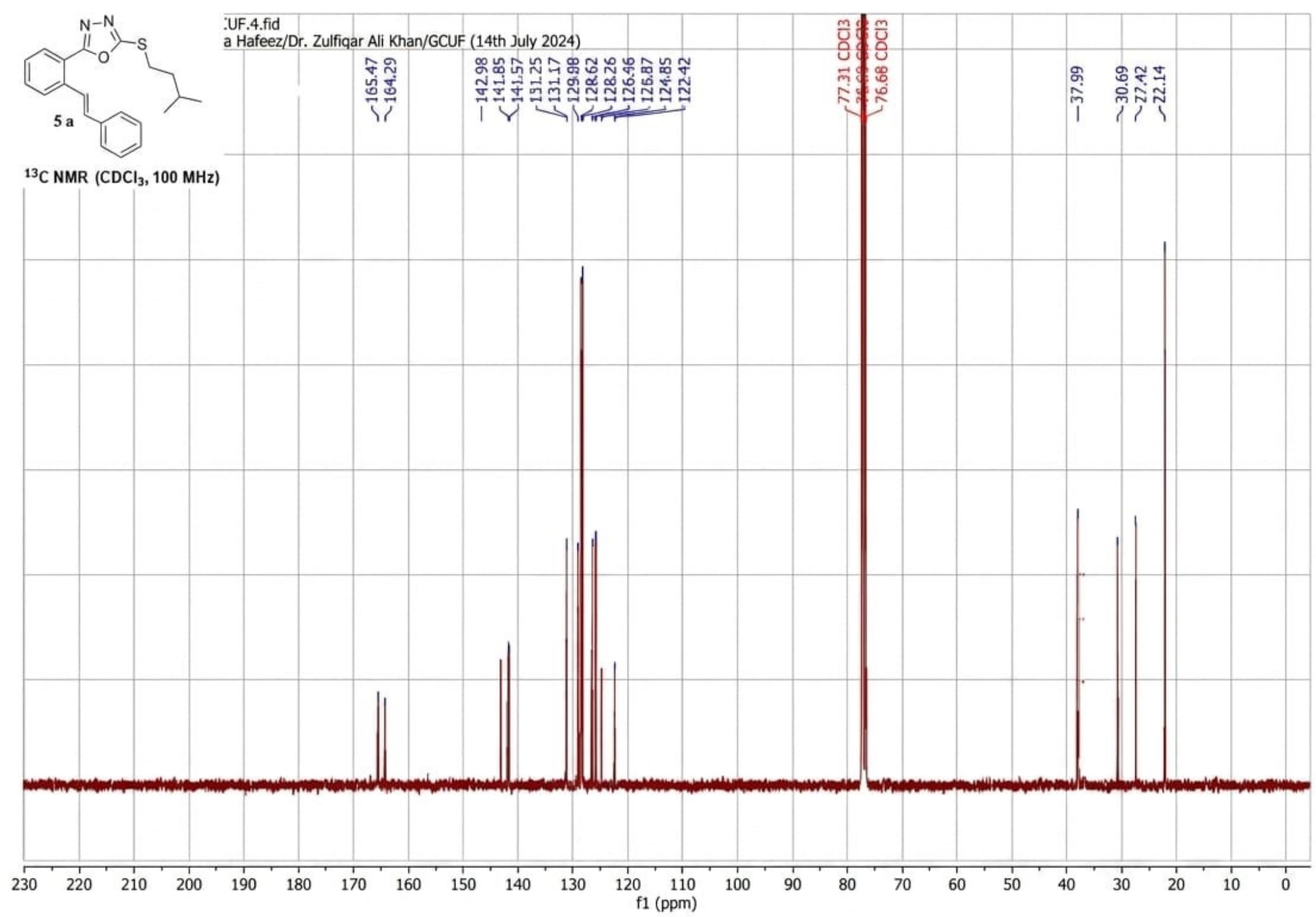

Fig. S4 FT-IR spectrum (KBr) of compound **5b**.

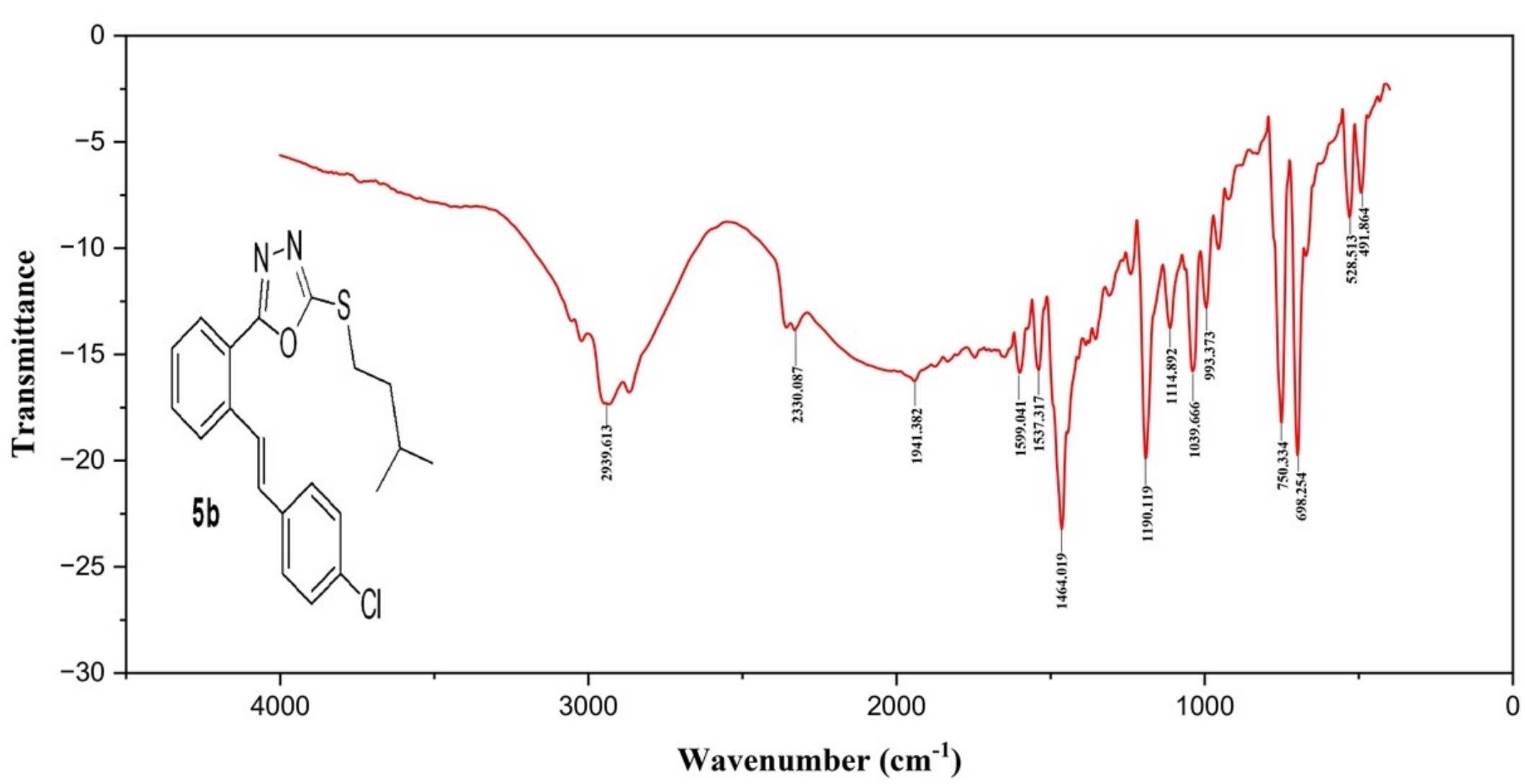

Fig. S5 ^1H NMR spectrum (400 MHz, $CDCl_3$) of compound **5b**.

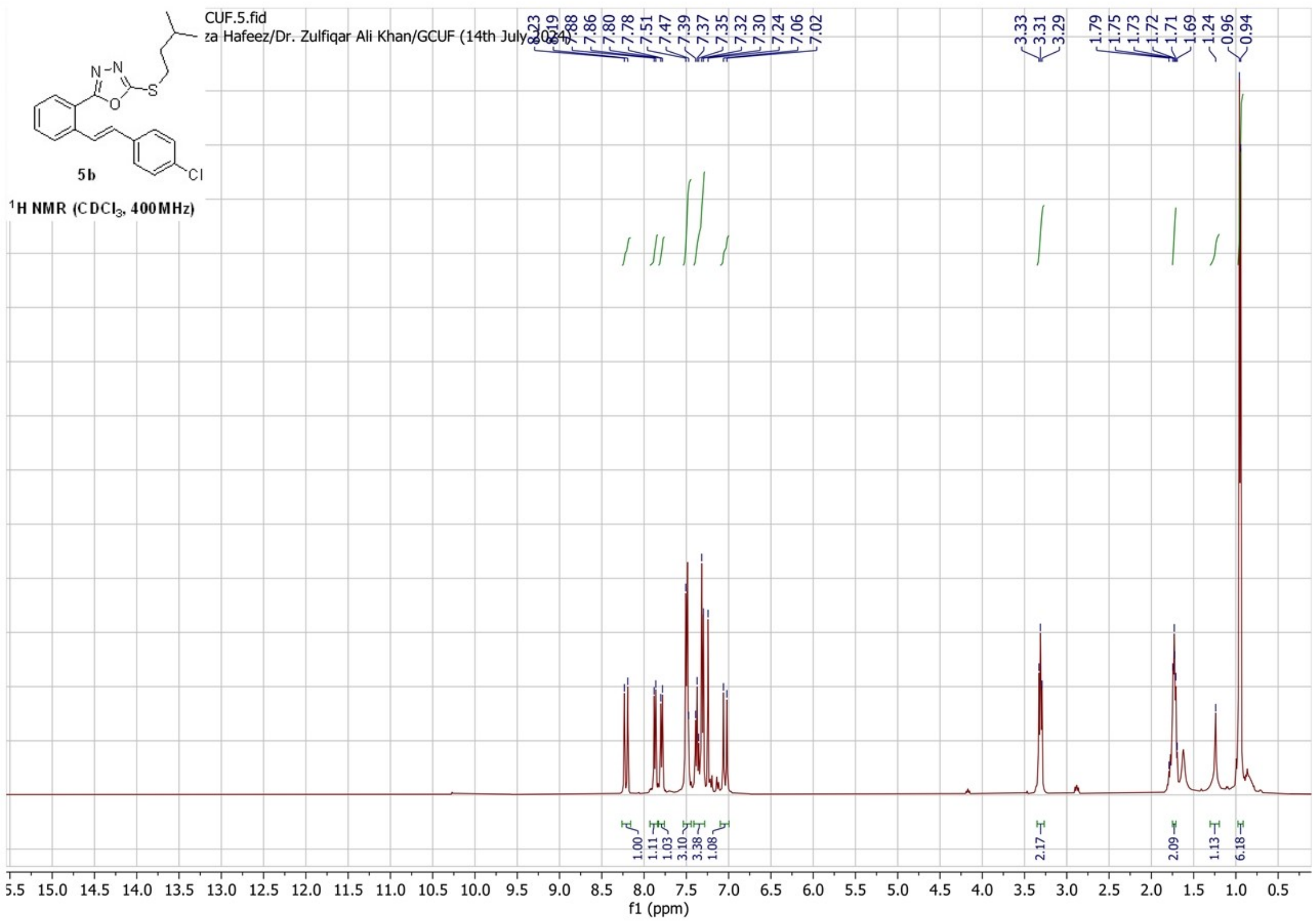

Fig. S6 ^{13}C NMR spectrum (100 MHz, $CDCl_3$) of compound **5b**.

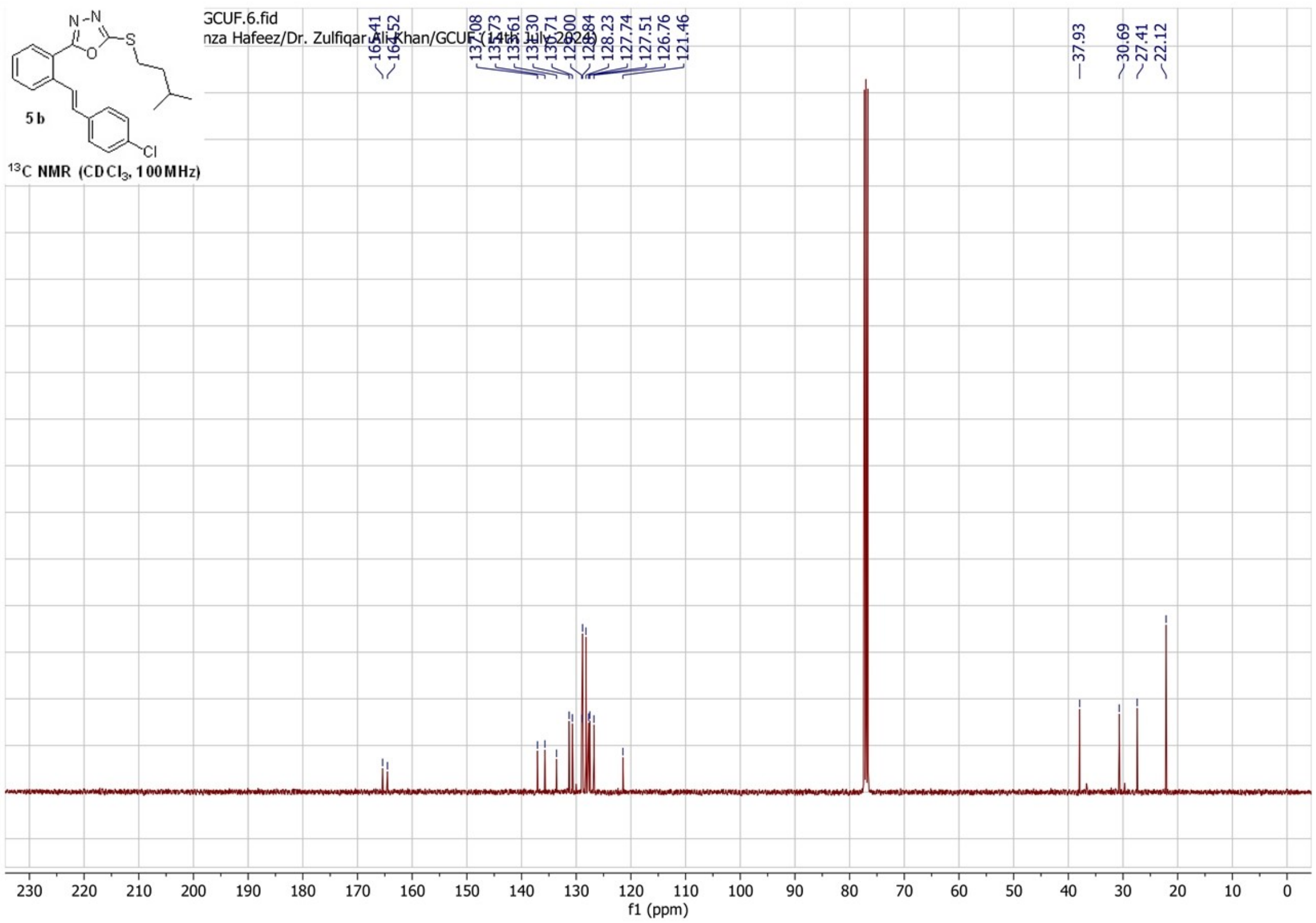

Fig. S7 FT-IR spectrum (KBr) of compound **5c**.

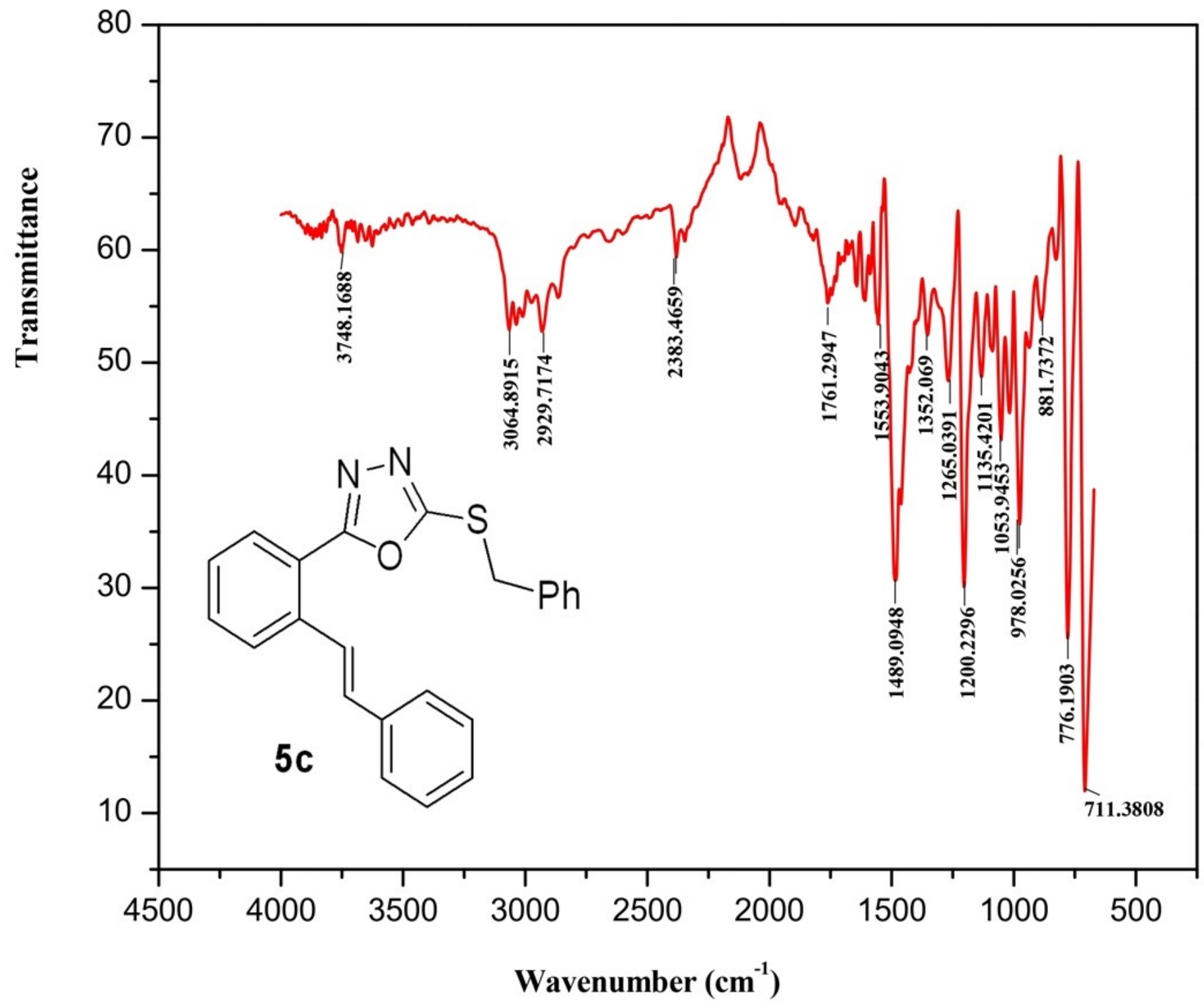

Fig. S8 ^{1}H NMR spectrum (400 MHz, $CDCl_3$) of compound **5c**.

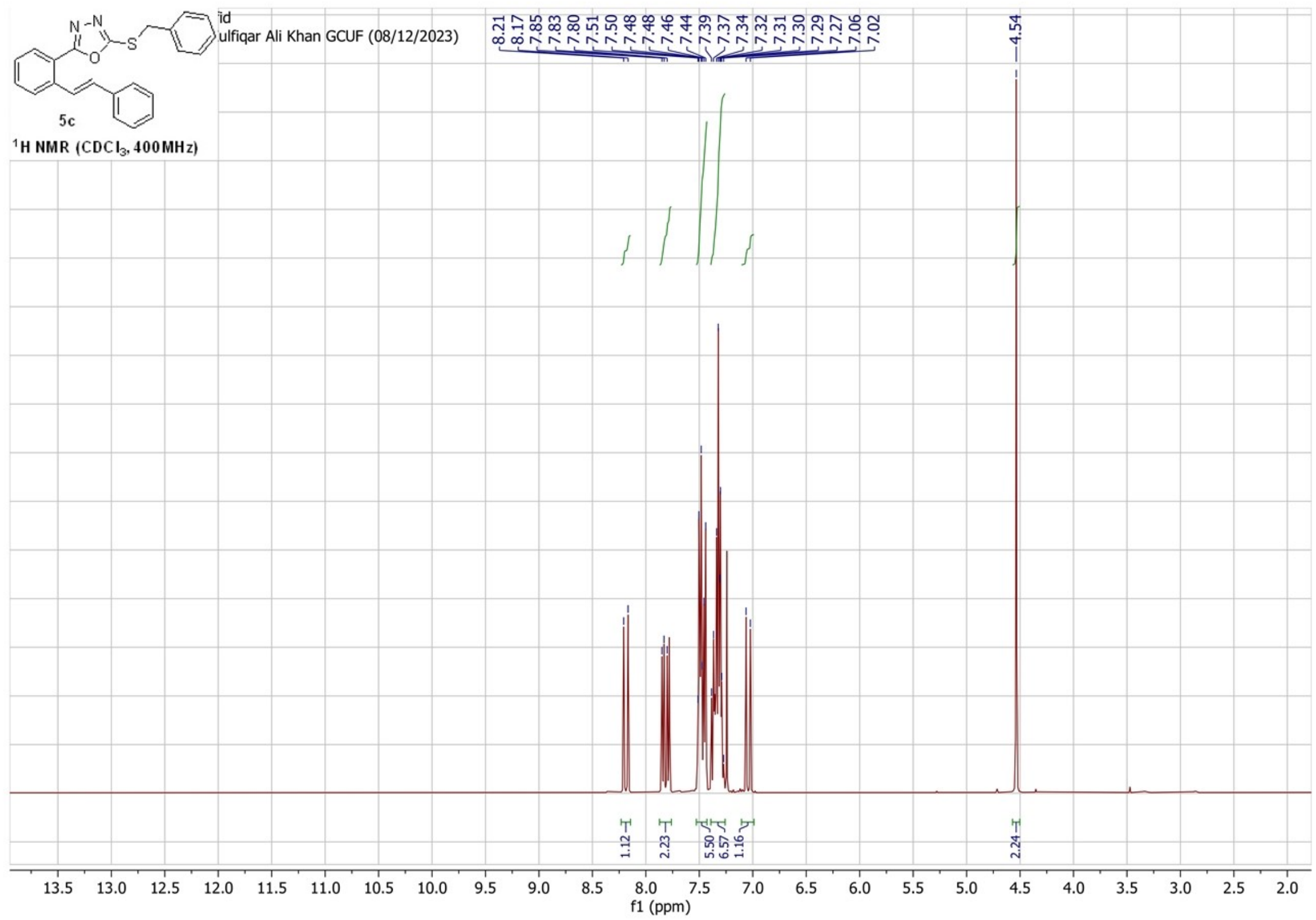

Fig. S9 ^{13}C NMR spectrum (100 MHz, $CDCl_3$) of compound **5c**.

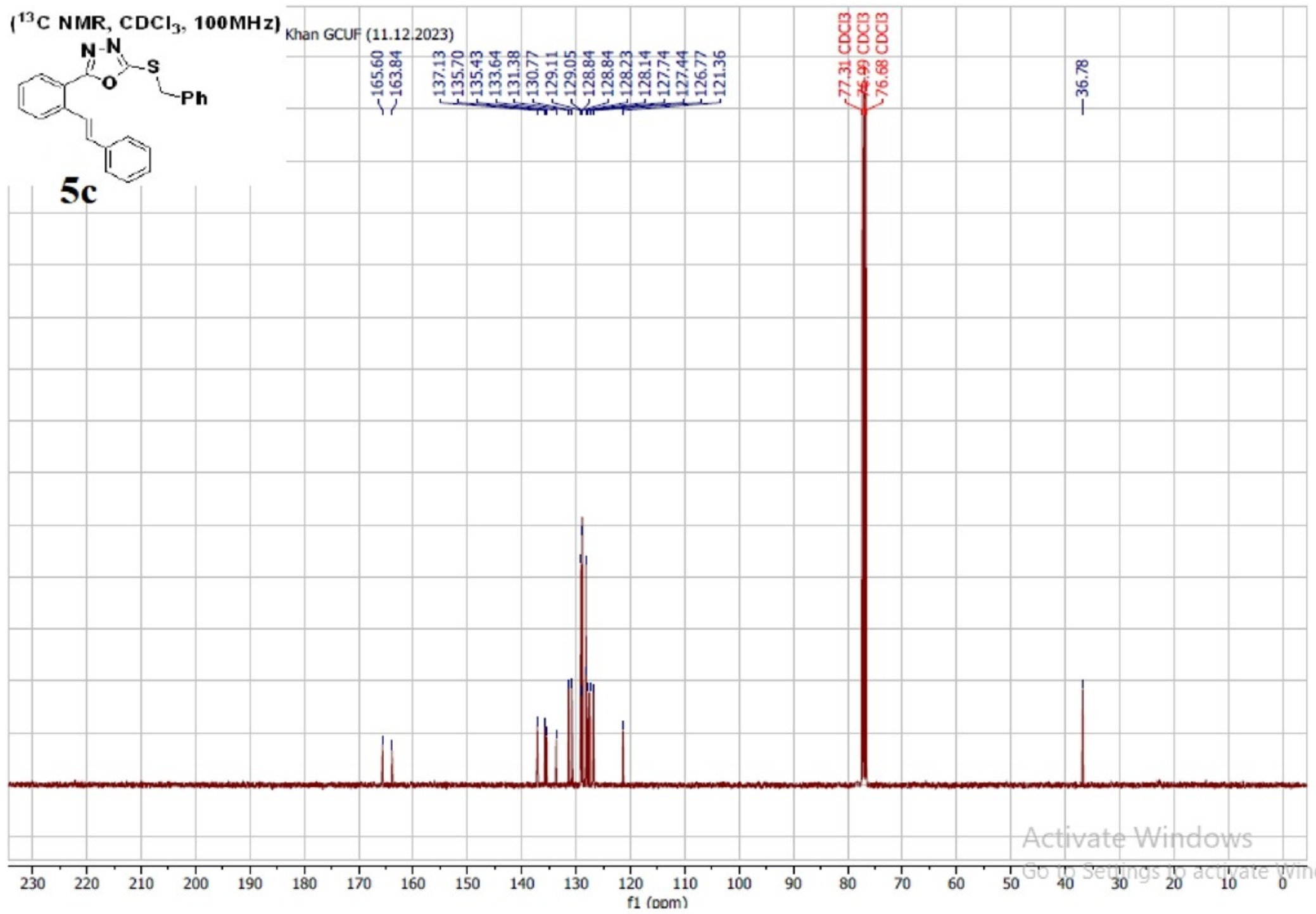

Fig. S10 FT-IR spectrum (KBr) of compound **5d**.

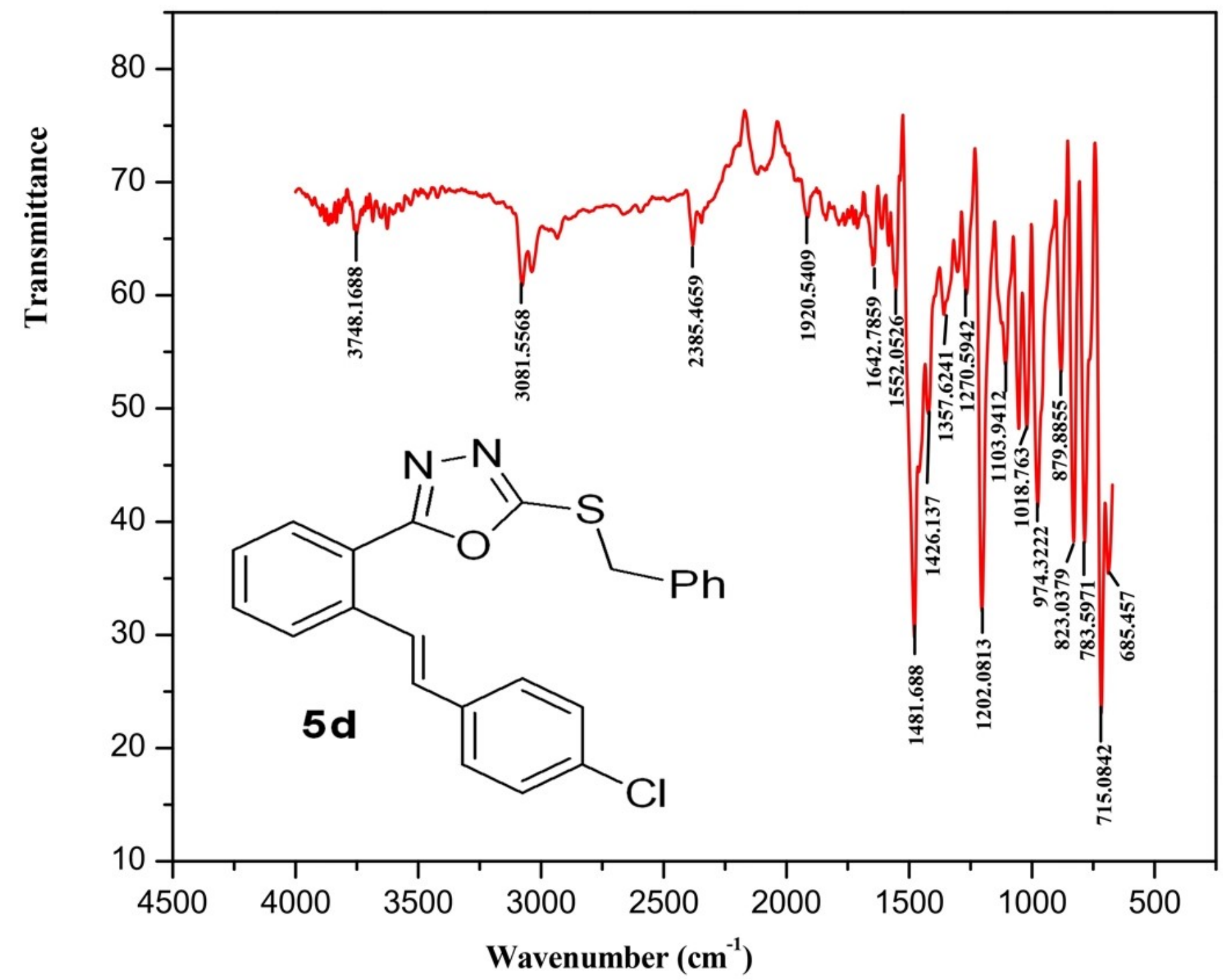

Fig. S11 ^{1}H NMR spectrum (400 MHz, $CDCl_3$) of compound **5d**.

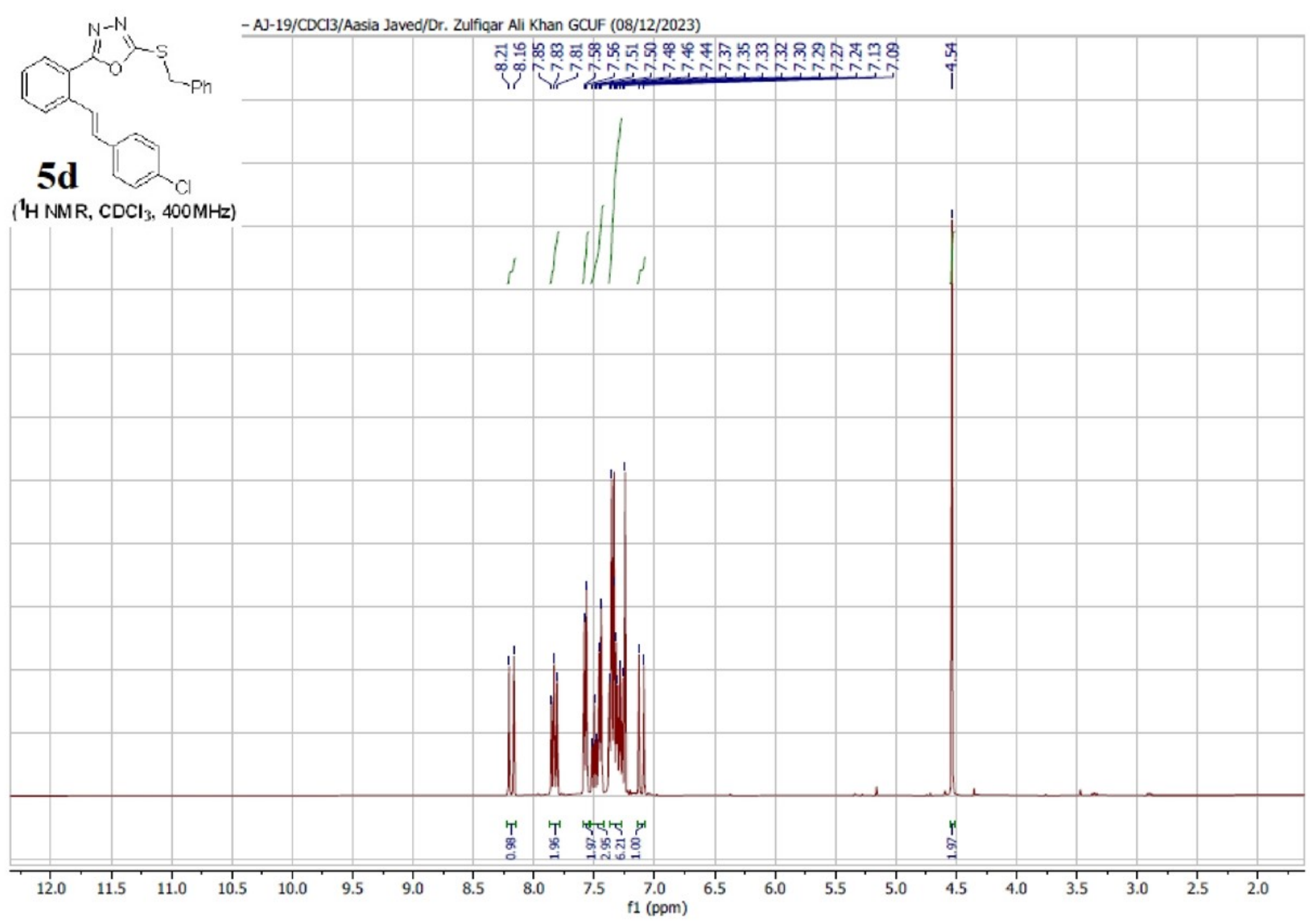

Fig. S12 ^{13}C NMR spectrum (100 MHz, $CDCl_3$) of compound **5d**.

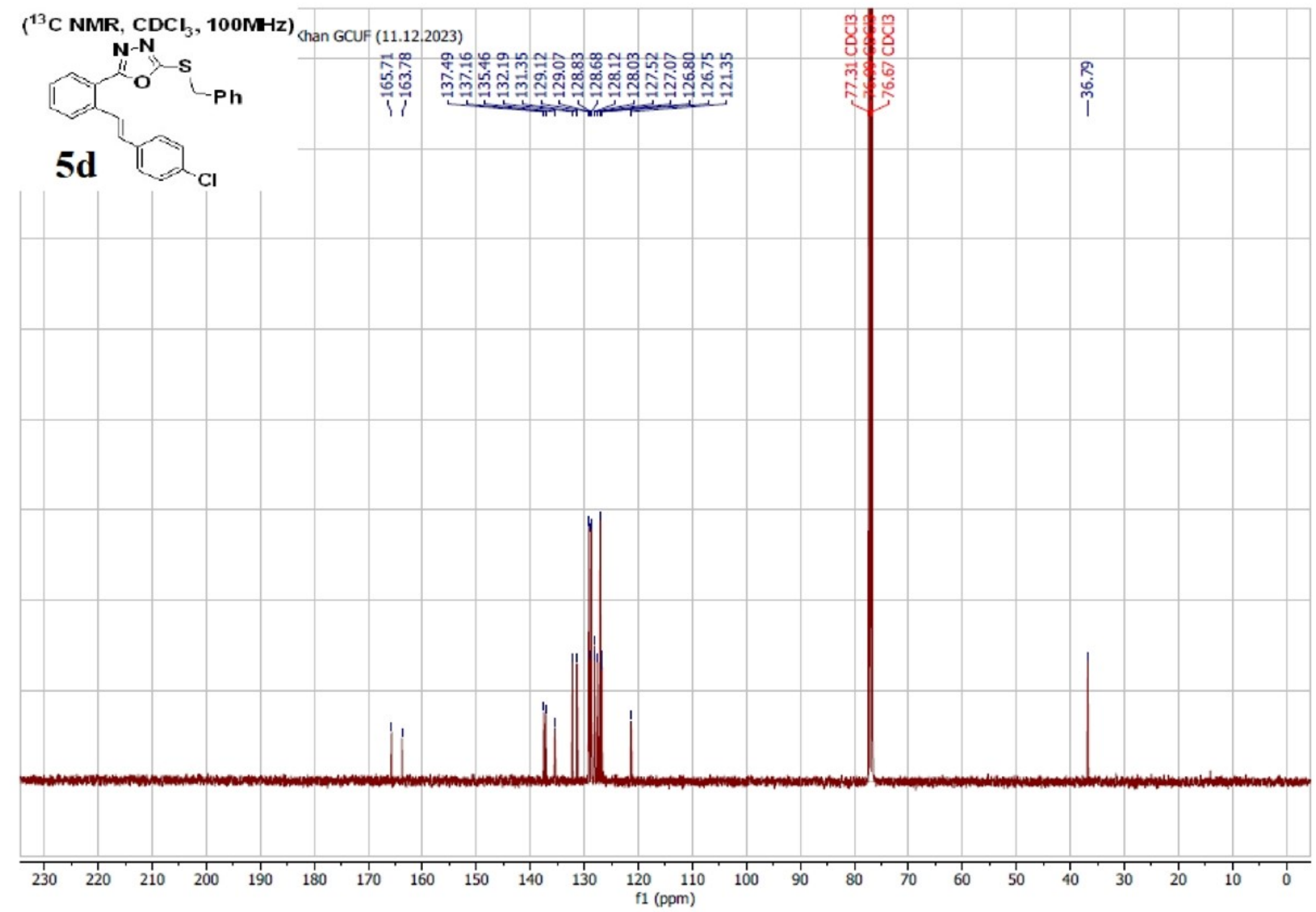

Fig. S13 FT-IR spectrum (KBr) of compound **5e**.

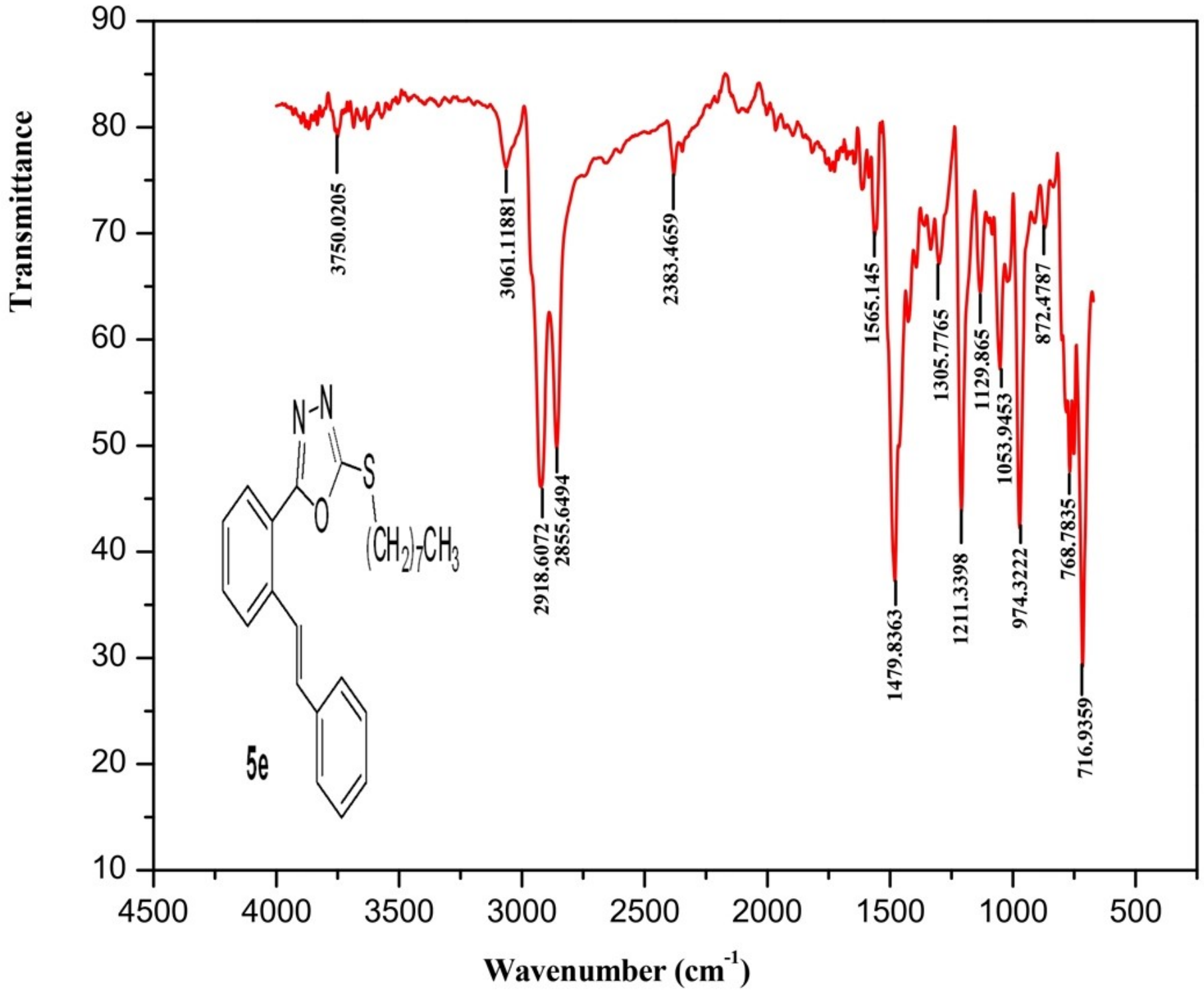

Fig. S14 ^{1}H NMR spectrum (400 MHz, $CDCl_3$) of compound **5e**.

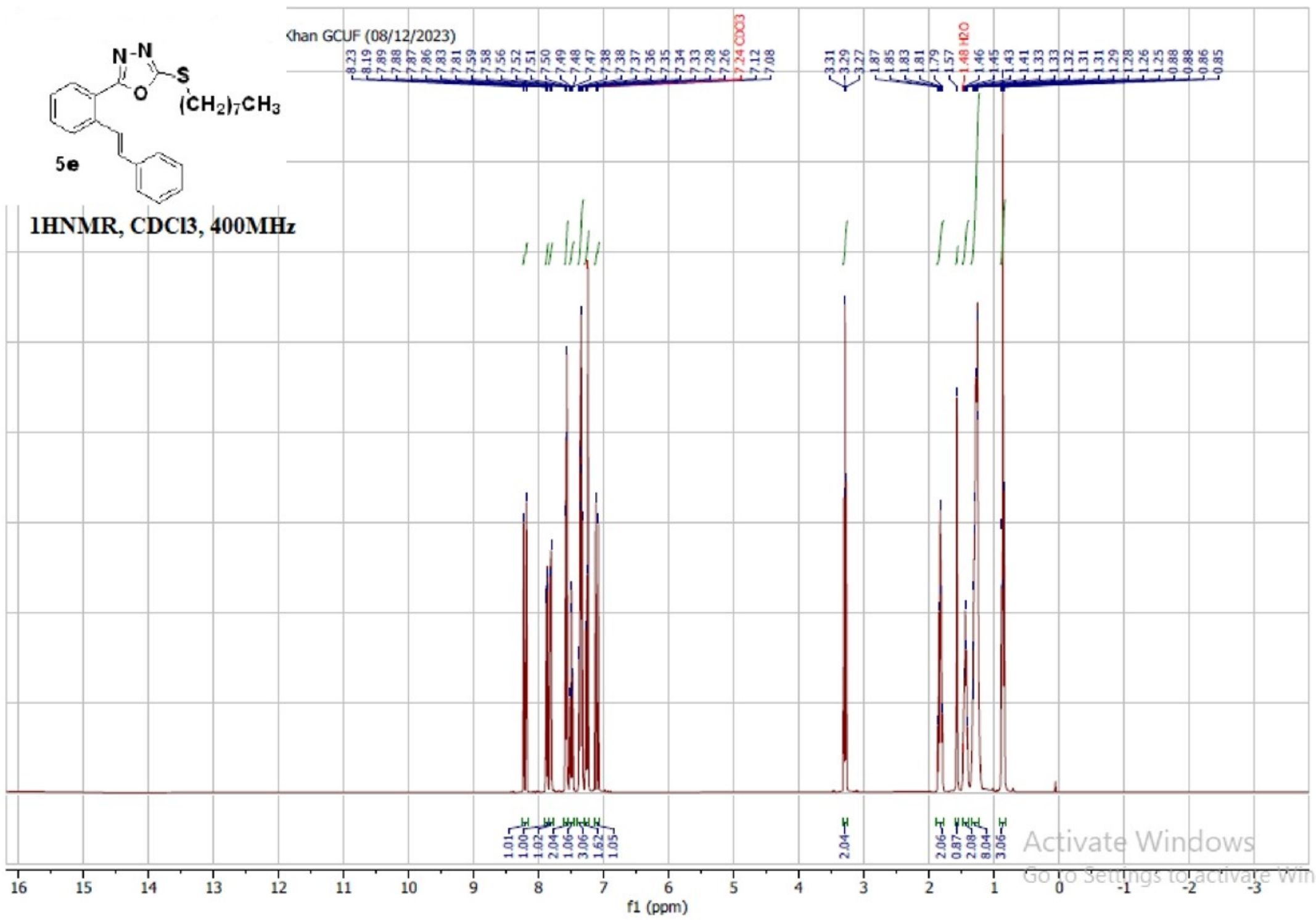

Fig. S15 ^{13}C NMR spectrum (100 MHz, $CDCl_3$) of compound **5e**.

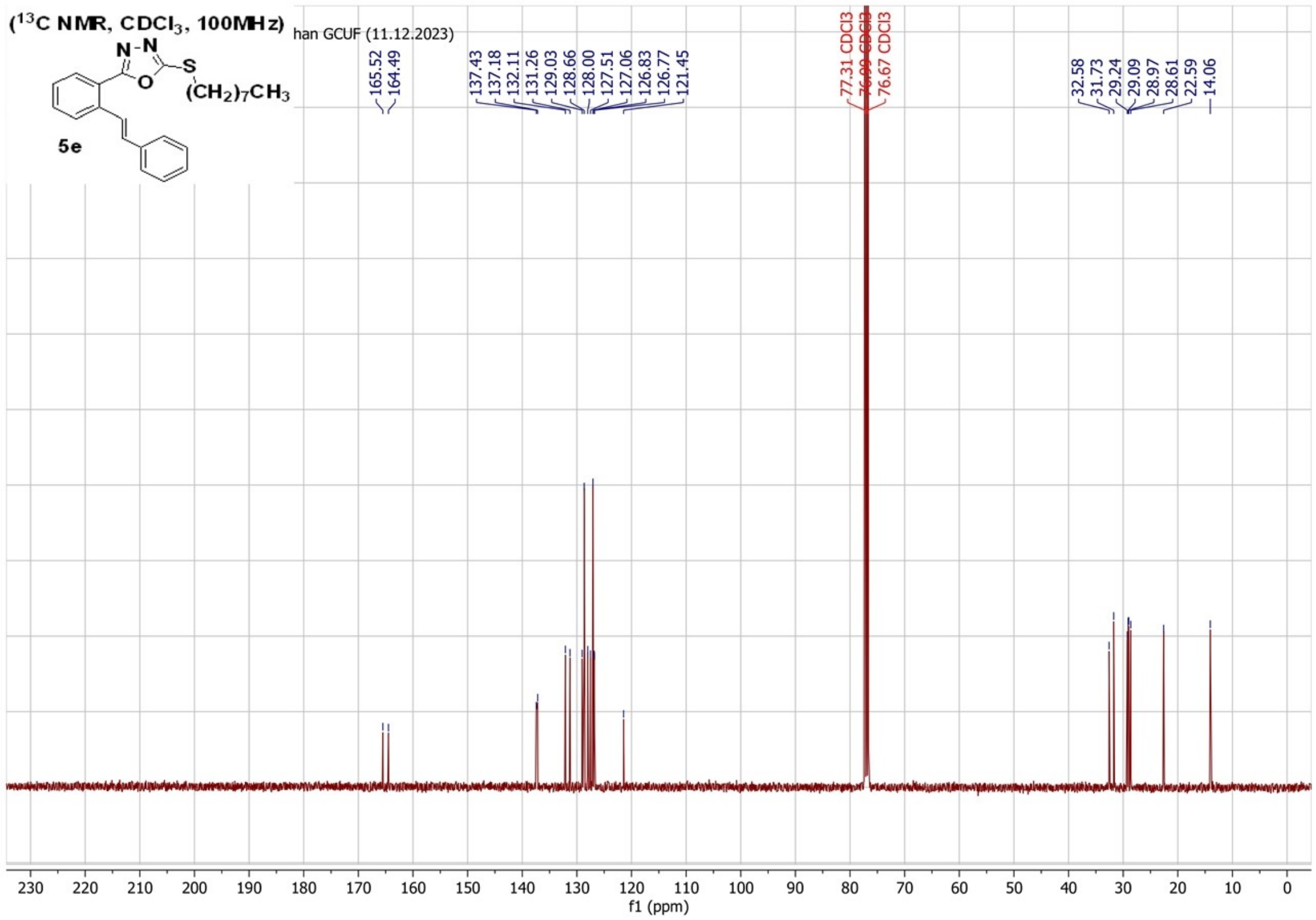

Fig. S16 FT-IR spectrum (KBr) of compound **5f**.

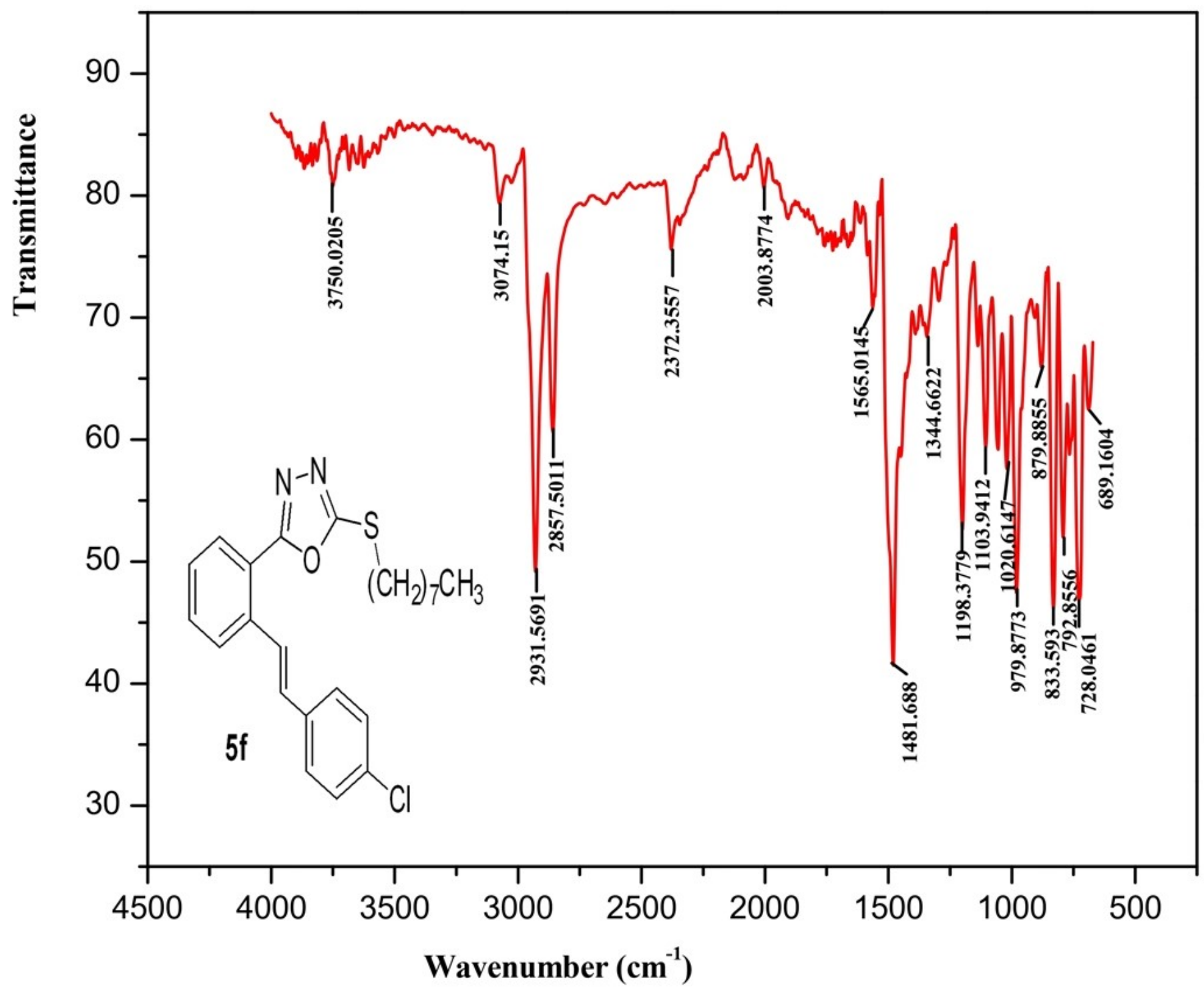

Fig. S17 ^{1}H NMR spectrum (400 MHz, $CDCl_3$) of compound **5f**.

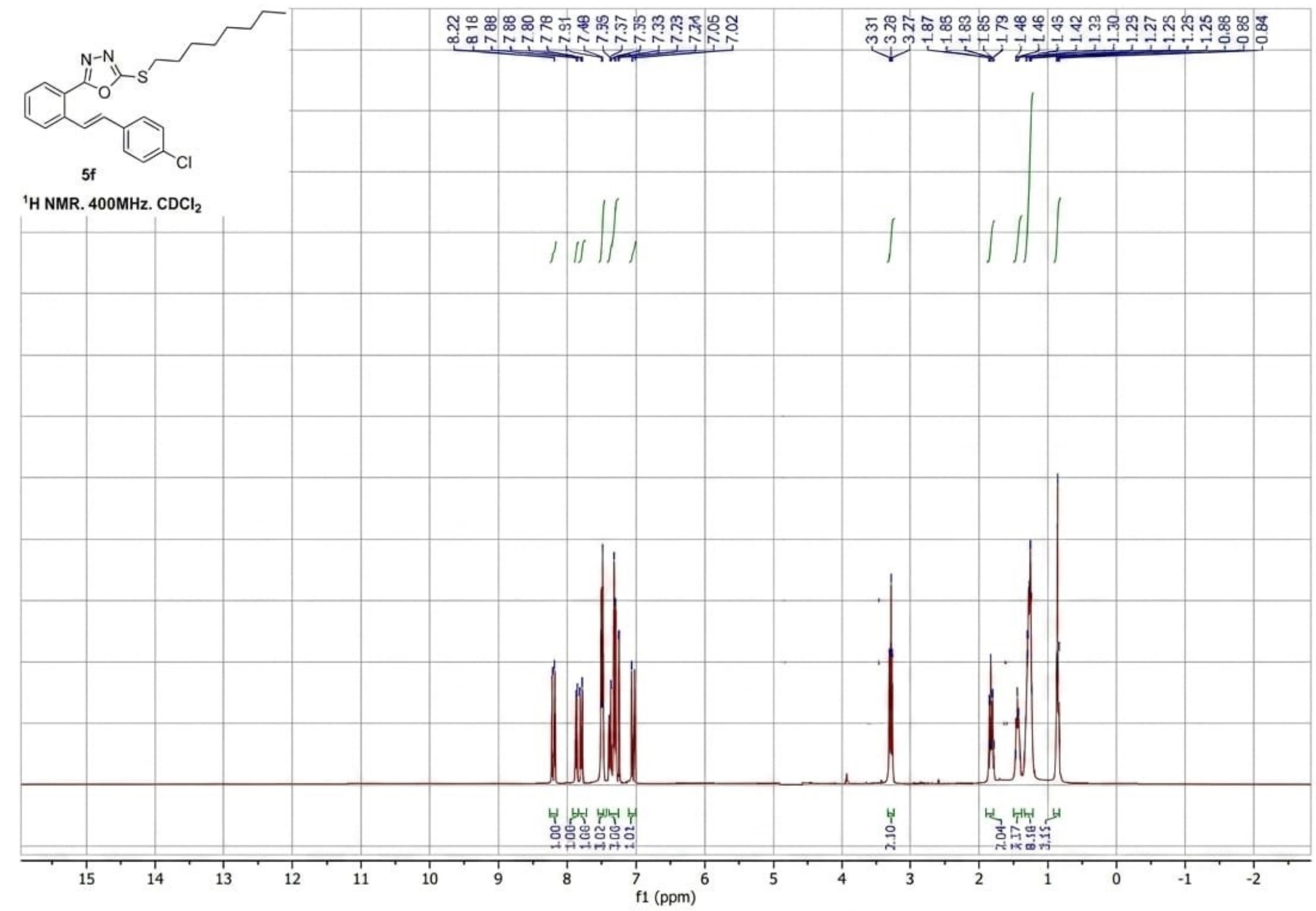

Fig. S18 ^{13}C NMR spectrum (100 MHz, $CDCl_3$) of compound **5f**.

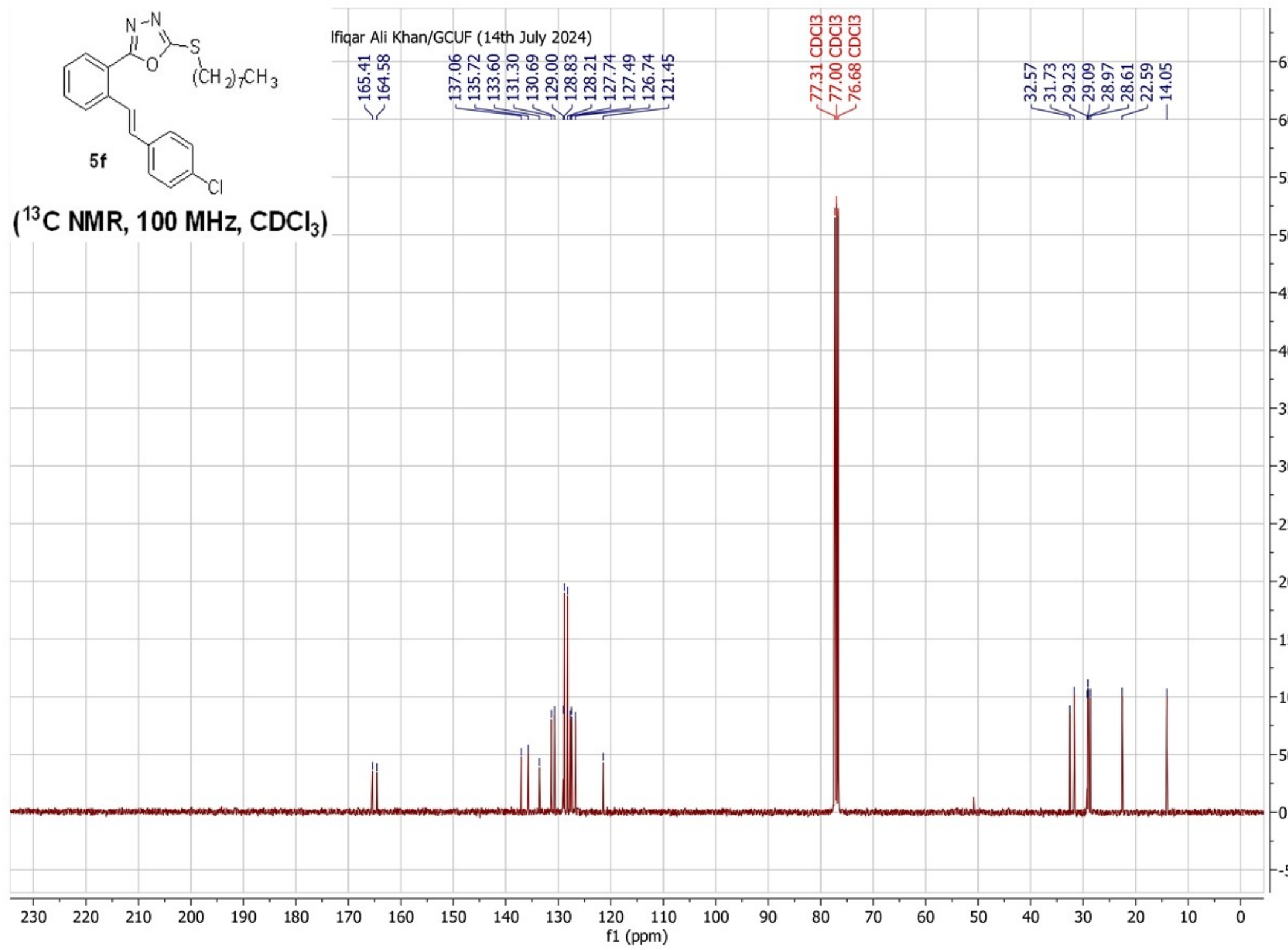

<< Insert spectrum image here >>
